# Life course BMI trajectories from childhood to mid-adulthood are differentially associated with anxiety and depression outcomes in middle age

**DOI:** 10.1038/s41366-023-01312-6

**Published:** 2023-05-09

**Authors:** Claire Gallagher, Jane Pirkis, Katrina A. Lambert, Jennifer L. Perret, Gulshan B. Ali, Caroline J. Lodge, Gayan Bowatte, Garun S. Hamilton, Melanie C. Matheson, Dinh S. Bui, Michael J. Abramson, E. Haydn Walters, Shyamali C. Dharmage, Bircan Erbas

**Affiliations:** 1 Centre for Epidemiology and Biostatistics, Melbourne School of Population and Global Health, University of Melbourne, Melbourne, VIC Australia; 2 Centre for Mental Health, Melbourne School of Population and Global Health, University of Melbourne, Melbourne, VIC Australia; 3 School of Psychology and Public Health, La Trobe University, Melbourne, VIC Australia; 4 Department of Basic Sciences, Faculty of Allied Health Sciences, University of Peradeniya, Peradeniya, Sri Lanka; 5 Monash Lung, Sleep, Allergy & Immunology, Monash Health, Melbourne, VIC Australia; 6 School of Clinical Sciences, Monash University, Melbourne, VIC Australia; 7 Population Health Solutions, Telstra Health, Melbourne, VIC Australia; 8 School of Public Health & Preventive Medicine, Monash University, Melbourne, VIC Australia; 9 School of Medicine, University of Tasmania, Hobart, VIC Australia

**Keywords:** Risk factors, Epidemiology, Obesity

## Abstract

**Background/Objective:**

Obesity is a risk factor for multimorbidity, including depression and possibly anxiety. However, it is currently unclear how patterns of change in BMI over the life course differentially influence the magnitude in risk of depression and anxiety in mid-adulthood. We aimed to examine associations between BMI trajectories from childhood to adulthood and the risk of depression and anxiety in middle age.

**Methods:**

In the Tasmanian Longitudinal Health Study (*n* = 2416), five distinct BMI trajectories were previously defined from age 5 to 45 years using group-based modelling. At age 53, current depression and anxiety were assessed using the Patient Health Questionnaire and the Generalized Anxiety Disorder scale, respectively. Logistic regression models adjusted for potential confounders estimated associations between BMI trajectories and these outcomes.

**Results:**

Those belonging to the child average-increasing (OR = 2.24; 95%CI: 1.24, 4.06) and persistently high (OR = 2.64; 1.26, 5.52) trajectories were more likely to have depression in middle age, compared to the persistently average trajectory. However, the odds of experiencing greater severity of depressive symptoms was highest in the child average-increasing group (OR = 2.36; 1.59, 3.49). Despite finding no evidence of association between BMI trajectories and current anxiety, we observed less severe symptoms in the child high-decreasing trajectory (OR = 0.68; 0.51, 0.91).

**Conclusion:**

We found an increased risk of depression in middle age among individuals with a persistently high BMI from childhood to mid-adulthood and individuals with an average BMI in childhood which then increased consistently throughout adulthood. Encouragingly, resolving childhood adiposity by adulthood was associated with lesser anxiety symptoms. Taken together, these findings highlight the need to target mental health screening and treatment towards high-risk BMI trajectory groups and the importance of early interventions to prevent and resolve excess weight.

## Introduction

Due to widespread community prevalence, depression and anxiety are now leading causes of global disability in adults, responsible for more than 40 million years of healthy life lost in 2020 [1]. The debilitating nature of these common mental disorders places substantial burden on individuals and their families, and costs the global economy US$1 trillion dollars each year in lost productivity and health care expenditure [2]. Accordingly, the prevention of mental disorders has become a global health priority, with growing interest in identifying the underlying risk factors for depression and anxiety at a young age and identifying possible target groups for early interventions.

The complex relationship between obesity and mental health has received increasing attention, with systematic reviews and meta-analyses finding that adults with obesity are at greater risk of subsequent depression [3] and possibly anxiety [4]. However, despite these findings, most of the research to date has considered obesity cross-sectionally, which fails to account for temporal changes in weight status and differences in the onset and duration of obesity exposure. Consequently, the longitudinal effects of obesity on long term mental health remain poorly understood and are of particular interest in light of global trends towards an increasingly earlier age of obesity onset [5].

Given the dynamic nature of weight status and the likely tracking of obesity from childhood to adulthood, research adopting a life-course perspective is necessary to appropriately quantify the effects of weight status on adult mental health and so, identify potentially sensitive periods during which changes in weight status might influence vulnerability to depression and anxiety. To date, only three studies have analysed trajectories of weight status over the life course in association with adult mental health, but all focused exclusively on depression [6–8]. Two [6, 7] of these studies modelled trajectories based on recalled body shape in non-population based samples and the other [8] modelled BMI trajectories only up to the age of 24 years.

To address these current knowledge gaps, population-based, prospective research extending BMI trajectories into mid-adulthood is needed. Especially considering that this age group experiences an increased vulnerability to, and burden of, mental disorders [9]. Furthermore, the association between life course BMI trajectories and adult anxiety has not been previously explored. Accordingly, the current study aimed to investigate the association between BMI trajectories from age 5 to 45 years and both current depression and anxiety risk at age 53 years, and whether these relationships were modified by sex, smoking, occupational class or alcohol intake.

## Methods

### Tasmanian Longitudinal Health Study

This study analyzed data from the Tasmanian Longitudinal Health Study (TAHS), a population-based, prospective community birth cohort study of children born in 1961, attending school in Tasmania, Australia in 1968 when baseline data collection occurred. Details regarding TAHS sampling procedures and methods of data collection have previously been described [10]. In summary, *n* = 8583 school children (age 7) underwent a clinical examination and questionnaire data were collected from their parents. Follow up surveys and/or examinations were conducted in 1974, 1979, 1991, 2002–2008 and 2012–2016 (*See* e-Methods in the online supplement for further details).

### Outcomes

#### Depression and anxiety

Depression and anxiety were measured at age 53 using the 9-item Patient Health Questionnaire (PHQ-9) and the 7-item Generalized Anxiety Disorder scale (GAD-7) [11, 12]. Current depression was defined as a PHQ-9 score ≥10 (sensitivity [Se]: 88% and specificity [Sp]: 88%) and the severity of depressive symptoms was defined using the recommended cut off scores: none (0–4), mild (5–9), moderate (10–14), moderately severe (15–19) and severe (≥20) [11]. Likewise, current anxiety was defined as GAD-7 score ≥10 (Se: 89% and Sp: 82%) and cut points of 5, 10 and 15 were used to represent mild, moderate and severe levels of anxiety symptoms, respectively [12]. We also assessed each individual symptoms as an individual outcome to explore whether different symptoms have different risk factors, as previously hypothesized [13]. (*See* e-Methods in the online supplement for further details).

### Exposure

#### Body mass index trajectories

**Fig. 1:**
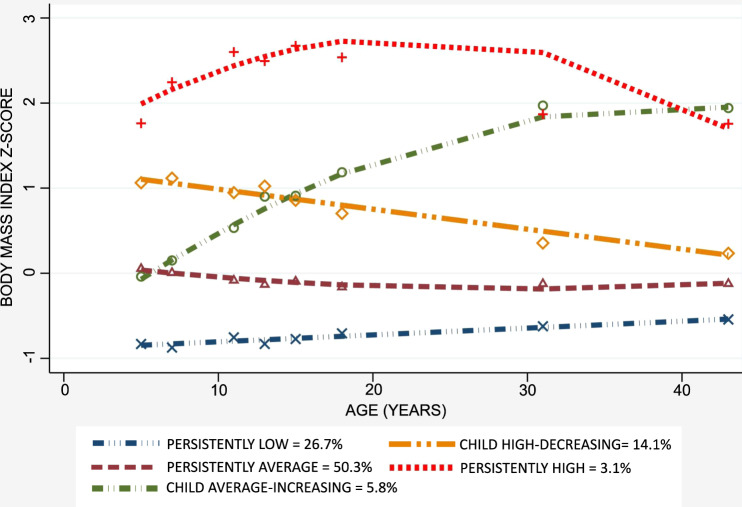
BMI z-score trajectories from age 5 to 43 years. Five BMI trajectories were identified using group-based trajectory modelling. Body mass index was measured at age 5, 7, 10, 13, 15, 20, 30 and 43 years, and each node in the graph represents the mean BMI z-score at each age. The persistently average trajectory comprised 50.3% of participants; the persistently low trajectory, 26.7%; the child high-decreasing trajectory, 14.1%; the child average-increasing trajectory, 5.8% and the persistently high trajectory, 3.1%.

Height and weight were measured at ages 7, 13 and 20, but self-reported by participants at ages 30 and 43. Additional measurements were extracted from school medical records at ages 5-6, 10-11 and 14-15 years. BMI was calculated as weight (kg)/height^2^ (m) and all eight measures were transformed into z scores standardized for sex. Five distinct BMI z-score trajectories from age 5 to 43 were estimated in the TAHS cohort using group-based trajectory modelling: *persistently average* (50.3%; *n* = 2109), *persistently low* (26.3%; *n* = 1120), *child high-decreasing* (14.1%; *n* = 592), *child average-increasing* (5.8%; *n* = 245), and *persistently high* (3.1%; *n* = 128) (Fig. 1) [14]. The modelling of BMI trajectories has previously been published [14] and further details are described in the online supplement. In brief, the number and shape of trajectories were determined by sequentially increasing the number of trajectories and decreasing the order of the polynomial until all functions were significant. The optimal model was then selected based on the Bayesian Information Criteria (BIC), group posterior probability and plausibility/clinical interpretation of trajectory shapes.

#### Other variables

Based on current knowledge, potential confounding variables were identified, and a directed acyclic graph (DAG) was developed to determine the minimally sufficient adjustment set (Supplementary Fig. S1). The following covariates measured at age 53 were adjusted for in the models: sex, education, occupational class, and smoking status. Additionally, the following variables measured at age 53 were selected as possible effect modifiers: sex, occupational class, smoking status and alcohol intake. See e-Methods in online supplement for further details.

### Statistical analyses

The characteristics of participants and the distribution of mental health outcomes across BMI trajectories were compared using the χ^2^ test for categorical variables and ANOVA or Welch’s ANOVA with Games-Howell post hoc tests for continuous variables, as appropriate. When analysing the distribution of anxiety symptoms and depressive symptoms, categories were combined to satisfy the assumptions of the χ^2^ test.

Binary and ordinal logistic regression analyses were conducted to estimate both unadjusted and adjusted odds ratios (OR) and 95% confidence intervals (95%CI) for the association between BMI trajectories and depression and anxiety outcomes, using the persistently average trajectory as the reference.

We explored sex, occupational class, smoking status and alcohol intake at age 53 as possible effect modifiers using interaction terms and likelihood ratio tests. Stratified analyses were presented when significant interactions were found, however we decided a priori to stratify all models by sex.

Analyses were conducted using STATA version 16 (StataCorp, College Station, TX) and SPSS version 23 (IBM Corp., Armonk, NY). A *p*-value <0.05 was considered as the level of significance for all tests except for the interaction terms, where a *p* value <0.1 was applied.

## Results

### BMI trajectory groups and membership characteristics

Participant characteristics across BMI trajectory groups are summarised in Table 1. The *persistently average* and *child high-decreasing* trajectories were predominantly male (*p* = 0.04). A greater proportion of participants in the *child average-increasing* and *persistently high* trajectories were from a lower occupational class (*p* = 0.01), but consumed low or no alcohol (*p* ≤ 0.001). BMI at 53 years also differed between trajectory groups (*p* < 0.001), with post-hoc analyses demonstrating that mean BMI differed between all groups except the *child average-increasing* and *persistently high* trajectories. The characteristics of participants lost to follow-up are presented in Supplementary Table S1. Whilst there was no significant difference in the proportion of BMI trajectory members, attrition was more likely in males (43.7% vs 38.0% of females) and participants with obesity (47.1% vs 39.3% normal/underweight and 39.5% overweight participants).

**Table 1:** Demographic, anthropometric, and behavioural characteristics of participants at 53 years across BMI trajectory groups.

Characteristics	Total Sample (*n* = 4194)	Persistently Average (*n* = 2109)	Persistently Low (*n* = 1120)	Child High-Decreasing (*n* = 592)	Child Average-Increasing (*n* = 245)	Persistently High (*n* = 128)	*P*-Value
Sex – Female % (*n*)	48.6% (2039)	47.2 % (995)	50.0% (560)	47.3% (280)	57.1% (140)	50.0% (64)	**0.04**^**a**^
Age (years) – mean (SD)	53.0 (0.9)	53.0 (0.9)	52.9 (0.9)	53.0 (0.9)	53.0 (0.9)	53.0 (0.9)	0.67^b^
Educational Attainment - % (*n*)							0.24^a^
Less than high school	32.5% (797)	32.8% (406)	33.0% (222)	28.0% (98)	37.5% (48)	33.3% (23)	
Yr. 12, certificate, diploma	43.2% (1060)	42.4% (525)	43.0% (289)	44.6% (156)	47.7% (61)	42.1% (29)	
University or post graduate degree	24.4% (599)	24.7% (306)	24.0% (161)	27.4% (96)	14.8% (19)	24.6% (17)	
*Occupational class - % (n)*							**0.01**^**a**^
Skill Level 1 (highest)	34.2% (836)	34.5% (425)	33.7% (225)	36.4% (127)	26.8% (34)	37.3% (25)	
Skill Level 2	14.2% (347)	14.0% (173)	15.3% (102)	15.5% (54)	10.2% (13)	7.5% (5)	
Skill Level 3	19.7% (482)	20.7% (255)	20.1% (134)	19.5% (68)	16.5% (21)	6.0% (4)	
Skill Level 4	18.3% (447)	17.7% (218)	18.4% (123)	16.3% (57)	22.8% (29)	28.9% (20)	
Skill Level 5	13.6% (332)	13.1% (162)	12.6% (84)	12.3% (43)	23.6% (30)	19.4% (13)	
*BMI – mean (SD)*	28.3 (5.5)	28.0 (4.5)	25.4 (3.8)	29.8 (4.7)	37.3 (6.2)	37.2 (8.1)	**<0.001**^**c**^
*Smoking Status -% (n)*							0.51^a^
Never	44.8% (1100)	44.2% (545)	46.4% (313)	42.9% (152)	43.0% (55)	50.7% (35)	
Former	38.5% (946)	38.6% (476)	38.7% (259)	37.3% (131)	44.5% (57)	33.3% (23)	
Current	16.7% (409)	17.1% (222)	15.3% (103)	19.4% (68)	12.5% (16)	15.9% (11)	
*Alcohol Intake - % (n)*							**<0.001**^**a**^
Non-drinkers	24.8% (613)	22.5% (279)	23.2% (158)	23.8% (84)	48.1% (62)	43.5% (30)	
<10 standard drinks per week	36.8% (909)	36.6% (455)	36.9% (251)	37.7% (133)	34.1% (44)	37.7% (26)	
≥10 standard drinks per week	38.5% (951)	40.9% (508)	39.9% (271)	38.5% (136)	17.8% (23)	18.8% (13)	

*BMI* body mass index. ^a^*p*-value derived from Chi^2^. ^b^*p*-value derived from ANOVA. ^c^*p*-value derived from Welch’s ANOVA. Bold text is used to highlight significant results.

### BMI trajectories in association with depression

The overall prevalence of current depression at age 53 years was 6.9% and was greatest in the *persistently high* trajectory (16.2%) and lowest in the *child-high decreasing* trajectory (5.7%, Table 2). However, these trends differed slightly among women, with prevalence rates highest in the *child average-increasing trajectory* (17.1%, Supplementary Table S2).

**Table 2:** Distribution of depression and anxiety in the total sample and across BMI trajectory groups.

	Total (*n* = 2416)	Persistently Average (*n* = 1212)	Persistently Low (*n* = 661)	Child High-Decreasing (*n* = 350)	Child Average-Increasing (*n* = 125)	Persistently High (*n* = 68)	*P*-Value
*Current Depression - n(%****)***							**<0.01**
Yes	166 (6.9%)	72 (5.9%)	46 (7.0%)	20 (5.7%)	17 (13.6%)	11 (16.2%)	
*Severity of Depressive Symptoms - n(%)*							**<0.01**^**a**^
Minimal	1872 (77.5%)	954 (78.7%)	520 (78.7%)	275 (78.6%)	75 (60.0%)	48 (70.6%)	
Mild	378 (15.7%)	186 (15.4%)	95 (14.4%)	55 (15.7%)	33 (26.4%)	9 (13.2%)	
Moderate	104 (4.3%)	51 (4.2%)	26 (3.9%)	13 (3.7%)	7 (5.6%)	7 (10.3%)	
Moderately severe	37 (1.5%)	11 (0.9%)	14 (2.1%)	5 (1.4%)	4 (3.2%)	3 (4.4%)	
Severe	25 (1.0%)	10 (0.8%)	6 (0.9%)	2 (0.6%)	6 (4.8%)	1 (1.5%)	
*Current Anxiety - n(%)*							0.35
Yes	199 (8.2%)	100 (8.2%)	52 (7.8%)	24 (7.0%)	16 (12.5%)	7 (10.3%)	
*Severity of Anxiety Symptoms - n(%)*							0.15^b^
Minimal	1756 (72.3%)	874 (71.8%)	489 (73.0%)	267 (77.4%)	81 (63.3%)	45 (66.2%)	
Mild	473 (18.5%)	243 (20.0%)	129 (19.3%)	54 (15.7%)	31 (24.2%)	16 (23.5%)	
Moderate	124 (5.1%)	63 (5.2%)	33 (4.9%)	14 (4.1%)	9 (7.0%)	5 (7.4%)	
Severe	75 (3.1%)	37 (3.0%)	19 (2.8%)	10 (2.9%)	7 (5.5%)	2 (2.9%)	

*BMI* body mass index. *P*-value derived from Chi^2^. ^a^categories “moderate”, “moderately severe” and “severe” were collapsed to satisfy the assumptions of the chi-square test. ^b^categories “moderate” and “severe” were collapsed to satisfy the assumptions of the chi-square test. Bold text is used to highlight significant results.

In adjusted models, the *child average-increasing* trajectory (OR = 2.24, 95%CI: 1.24, 4.06) and the *persistently high* trajectory (OR = 2.64, 95%CI: 1.26, 5.52) were at a greater risk of current depression at age 53, compared with the *persistently average* group (Table 3; unadjusted models in supplementary table S6). However, the odds of experiencing a greater severity of depressive symptoms were highest in the *child average-increasing* trajectory (OR = 2.36, 95%CI: 1.59, 3.49). This group was also more likely to experience each depressive symptom at an increased frequency (Supplementary Table S8). In comparison, the *persistently high* trajectory was more likely to experience appetite changes, whilst the *persistently low* trajectory was less likely to. The *child high-decreasing* trajectory was less likely to experience depressed mood and sleep disturbances.

**Table 3:** Multivariable binary and ordinal logistic regression exploring the association between BMI trajectory groups and depression and anxiety outcomes at age 53.

	Persistently low (*n* = 615)		Child high-decreasing (*n* = 330)		Child average-increasing (*n* = 125)		Persistently high (*n* = 68)	
	OR (95%CI)	*P*	OR (95%CI)	*P*	OR (95%CI)	*P*	OR (95%CI)	*P*
*Current Depression*	1.19 (0.80, 1.77)	0.39^a^	0.79 (0.46, 1.38)	0.41^a^	2.24 (1.24, 4.06)	**<0.01**^a^	2.64 (1.26, 5.52)	**0.01**^a^
*Severity of Depressive Symptoms*								
Minimal Mild Moderate Moderately Severe Severe	1.05 (0.83, 1.33)	0.69^b^	0.92 (0.68, 1.24)	0.57^b^	2.36 (1.59, 3.49)	**<0.01**^b^	1.61 (0.92, 2.82)	0.09^b^
*Current Anxiety*	0.99 (0.69, 1.42)	0.97^a^	0.78 (0.48, 1.26)	0.31^a^	1.56 (0.88, 2.77)	0.13^a^	1.24 (0.54, 2.83)	0.61^a^
*Severity of Anxiety Symptoms*^*b*^								
Minimal Mild Moderate Severe	0.95 (0.77, 1.18)	0.65^b^	0.68 (0.51, 0.91)	**0.01**^b^	1.38 (0.94, 2.03)	0.10^b^	1.10 (0.65, 1.88)	0.72^b^

*BMI* body mass index. ^a^binary logistic regression. ^b^ordered logistic regression. The reference category is the persistently average trajectory group. Adjusted for sex, educational attainment, occupational status, smoking status. Bold text is used to highlight significant results.

In sex-stratified models, the risk of depression was greater among males in the *persistently high* trajectory (OR = 3.68, 95%CI: 1.13, 12.0) and among women in the *child average-increasing* trajectory (OR = 2.77, 95%CI: 1.38, 5.58, Table 4). However, interaction terms were not significant. Regarding symptoms of depression, women in the *persistently low* trajectory were more likely than men to experience fatigue (OR = 1.29, 95%CI: 1.00, 1.67, p_int_ < 0.01; Supplementary Table S9).

**Table 4:** Multivariable binary and ordinal logistic regression exploring the association between life course BMI trajectory groups and mental health outcomes at age 53 stratified by sex.

	Persistently Low (*n* = 661)		Child High-Decreasing (*n* = 350)		Child Average-increasing (*n* = 125)		Persistently High (*n* = 68)		
	Female (*n* = 328)	Male (*n* = 333)	Female (*n* = 189)	Male (*n* = 161)	Female (*n* = 76)	Male (*n* = 49)	Female (*n* = 42)	Male (*n* = 26)	LTR *p*-value
*Current Depression*^*a*^	1.36 (0.82,2.28)	0.98 (0.51,1.86)	0.85 (0.43,1.69)	0.66 (0.25,1.74)	2.77** (1.38,5.58)	1.33 (0.38,4.63)	2.26 (0.87,5.86)	3.68* (1.13,12.0)	0.77
*Severity of Depressive Symptoms*^*b*^									
Minimal Mild Moderate Moderately Severe Severe	1.13 (0.82,1.57)	0.96 (0.67,1.37)	0.85 (0.57,1.28)	0.99 (0.63, 1.56)	2.51*** (1.53,4.11)	2.16* (1.13,4.14)	1.42 (0.70,2.88)	1.94 (0.77,4.87)	0.89
*Current Anxiety*^a^	1.06 (0.67,1.66)	0.88 (0.48,1.59)	0.69 (0.37,1.29)	0.85 (0.39,1.89)	1.63 (0.82,3.25)	1.29 (0.43,3.86)	0.95 (0.32,2.83)	1.94 (0.54,6.93)	0.89
*Severity of Anxiety Symptoms*^b^									
Minimal Mild Moderate Severe	1.11 (0.83, 1.48)	0.80 (0.57, 1.09)	0.80 (0.55, 1.16)	0.51** (0.32, 0.83)	1.35 (0.83, 2.22)	1.47 (0.79,2.74)	0.82 (0.40, 1.68)	1.69 (0.75,3.78)	0.20

*BMI* body mass index. ^a^binary logistic regression, ^b^ordered logistic regression. The reference category is the persistently average trajectory group. *<0.05; **<0.01; ***<0.001. Bold text is used to highlight significant results in stratified models. Sex interaction terms were not significant (<0.1) in any of the models. Adjusted for educational attainment, occupational status, smoking status. LRT P value is the p-value of the likelihood ratio tests.

In effect-modification analyses, a greater risk of depression was observed among participants in both the *child average-increasing* and *persistently high* trajectories if they were from a lower occupational class (Supplementary Table S10). We found no evidence of effect modification by alcohol intake or smoking.

### BMI trajectories in association with anxiety

The overall prevalence of current anxiety was 8.2% and did not significantly differ across trajectories (Table 2). Although, the prevalence of anxiety in *child high-decreasing* trajectory was lower than average.

In adjusted models, BMI trajectories were not associated with current anxiety at age 53 years (Table 3; unadjusted models in supplementary table S6). However, the *child high-decreasing* trajectory was more likely to experience a lower severity of symptoms compared to the *persistently average* trajectory (OR = 0.68, 95%CI: 0.51,0.91). Furthermore, the *child average-increasing* trajectory was more likely to experience irritability (OR = 1.86, 95%CI: 1.31, 2.64) and feelings of apprehension (OR = 1.67, 95%CI: 1.07, 2.59), whilst a positive trend was observed for feeling nervous/anxious (OR = 1.38, 95%CI: 0.96, 1.97, *p* = 0.08) and uncontrollable worrying (OR = 1.44, 95%CI: 0.98, 2.11, *p* = 0.06; Supplementary Table S8)

In sex-stratified models, males in the *child high-decreasing* trajectory (OR = 0.51 95%CI: 0.32, 0.83) were less likely to experience a greater severity of anxiety symptoms (Table 4). When symptoms were analyzed individually, women in the *persistently low* and *child-high decreasing* trajectories experienced trouble relaxing significantly more frequently than men (p_int_=0.03 and 0.1, respectively) and females in the *persistently high* BMI trajectory experienced feelings of apprehension less frequently than men (p_int_=0.07; Supplementary Table S9).

In effect-modification analyses, a greater risk of anxiety was observed among participants in the *persistently low* BMI trajectory within strata of alcohol consumers (p_int_<0.1). Whereas a decreased risk of anxiety was observed within strata of lower occupational class (p_int_<0.1). We found no evidence of effect modification by smoking.

## Discussion

In our long-term population-based cohort, five distinct trajectories of BMI from ages 5 to 43 years were differentially associated with depression and to a lesser extent, anxiety at age 53. Specifically, the *persistently high* and *child average-increasing* trajectories were associated with a greater risk of current depression in mid-adult life, with participants in the *child average-increasing* trajectory more likely to experience a greater severity of symptoms. Furthermore, despite finding no association between BMI trajectories and the risk of current anxiety per se, participants in the *child high-decreasing* trajectory were less likely to experience severe symptoms of anxiety.

Current evidence on the longitudinal effects of life course obesity on depression in adulthood is limited [6–8] and only one other study has modelled BMI trajectories using prospectively collected data and only to early adulthood [8]. Consistent with what we observed in the *child average-increasing* trajectory, Perry et al. [8] found a greater risk of depression in young adults (24 years) who had a major increase in BMI following puberty. However, unlike our study, no greater risk of depression was found in the persistently high trajectory, possibly owing to differences in follow up duration (24 vs 43 years), cohort age (1991 vs 1961 birth cohort) and age at outcome assessment (early- vs. mid-adulthood).

Although less comparable, the studies that analyzed trajectories of recalled body size were conducted in specific sub-populations, namely middle aged university graduates [7] and post-menopausal women [6]. Sayon-Orea et al. [7] found that women with a large body size in childhood, which increased throughout adulthood, were at an increased risk of new-onset depression at age 40, but found no associations in males. Similarly, Perquier et al. [6] showed that a persistently large body size was associated with a greater risk of new onset depression post-menopause (age 65). And a marked increase in body size following puberty was associated with current depression post-menopause. When taken together, it seems excess weight that persists from childhood to adulthood, and excess weight gained across the adolescent transition, may increase vulnerability to depression in adulthood. This highlights the need to increase mental health screening and support among individuals with comparable weight trajectories.

Notably, our study extends the literature by demonstrating that in comparison to the *persistently high* BMI trajectory, belonging to the *child average-increasing* trajectory was associated with a greater risk of having more severe and frequent symptoms of depression and a greater risk of several anxiety symptoms. Although we would expect to see a dose-response relationship, where a greater duration of obesity worsens mental health, in actual fact it appears that participants belonging to the *child average-increasing* trajectory had the worst mental health profile. These novel insights suggest adolescence may be a period of increased sensitivity, during which, the onset of obesity has an apparent influence on long term mental health. The timely introduction of obesity prevention strategies during these formative years may aid in reducing the burden of depression. However, we cannot exclude the possibility that depression onset preceded weight gain in this group, as a bidirectional relationship is quite possible; [15] furthermore, earlier-onset of depression has also been associated with greater severity [16].

Although we couldn’t establish a statistically significant association between BMI trajectories and current anxiety, we did observe a positive trend in the two highest BMI trajectories. Despite limited longitudinal evidence on this topic, a meta-analysis of four prospective studies found a small, positive association between obesity and anxiety [4] and so it is possible that our study was insufficiently powered to pick up a small effect. This would not be surprising given the frequent clinical concurrence of anxiety and depression.

Encouragingly, the *child high-decreasing* trajectory was associated with a reduced likelihood of experiencing severe anxiety symptoms, sleep disturbance or depressed mood, and had no association with current depression. Previous studies have also found no association between excess weight confined to childhood only and adult depression risk; [8, 17–19] however, our study is the first to show resolving excess weight may promote mental health. These findings suggest that the potentially depressive effects of obesity may be reversible with its resolution and therefore amenable to intervention: highlighting the need for and value of early intervention strategies to prevent and treat excess weight. Moreover, as the reference trajectory maintained a lower BMI throughout the duration of the observation period, it is likely that methods of weight reduction such as exercise and other healthy lifestyle behaviours partially explain the results observed in the *child high-decreasing* trajectory [20, 21]. And so, further research should explore methods of weight reduction in at-risk trajectory groups as possible intervention strategies to promote mental health and wellbeing.

To date, several plausible mechanisms have been suggested to explain why obesity may increase vulnerability to depression and anxiety. From a psychosocial perspective, weight-based stigmatization and discrimination faced by individuals with obesity have been shown to mediate associations between weight status, anxiety and depression, by increasing body image dissatisfaction, psychological distress, and lowering self-esteem [22]. Several related behaviours also offer explanatory pathways, as poor dietary patterns, physical inactivity, and sedentary lifestyles are common among individuals with obesity and present as risk factors for depression and anxiety [23]. More recently, increasing research attention has focused on underlying physiological and genetic mechanisms. Most prominently, chronic low-grade systematic inflammation, induced by obesity, has been identified as a key catalyst for depression, and possibly anxiety, by triggering dysregulation of the hypothalamic-pituitary-adrenal axis, with activated immune-inflammatory pathways inducing neuroendocrine abnormalities [24].

Sex differences were apparent in our stratified analyses; however, evidence of effect modification was weak. We observed an increased risk of depression among women in the *child average-increasing* trajectory, which is consistent with the findings of Perry et al. [8]. However, our study is the first to report a greater risk of depression among males in the *persistently high* trajectory. Although obesity and depression are more prevalent in women, sex differences in the relationship between obesity and depression are lesser known. It has been shown that women experience greater body dissatisfaction and place greater value on their appearance regardless of age [25]. In this context, a consistently increasing BMI might be negatively affecting body image among women in the *child average-increasing* trajectory, causing an increased vulnerability to depression. Somewhat paradoxically, however, a greater magnitude in the risk of depression was observed among males in the *persistently high* trajectory. Although this finding is unexpected, it’s possible that women in this group have sought support for their mental health, as males are less likely to seek treatment [26].

We found evidence of effect modification by socioeconomic status, such that a lower occupational class predicted a greater risk of depression in the *persistently high* trajectory. Given the disadvantages faced by those from lower socioeconomic backgrounds, factors such as food insecurity, financial and social stress, and reduced health care access, may explain this association. However, suprisingly, participants in the *persistently low* trajectory were less likely to report anxiety if they were from a lower occupational class; and the reason for this finding is not well understood. Furthermore, we also found a greater risk of anxiety among participants in the *persistently low* trajectory if they were consumers of alcohol, suggesting alcohol screening may be pertinent in this group. However, further research is needed to replicate our findings.

### Strengths and limitations

The main strength of this study is that our BMI trajectories were modelled using eight repeated measures of BMI, with five collected across the transition from childhood to adulthood. This enabled us to adequately characterize within-subject variations in obesity over the life course. Furthermore, data were prospectively collected from a large representative sample of the Tasmanian population, and we assessed a variety of mental-health outcomes to understand the point prevalence, severity and symptomatology of anxiety and depression.

Several limitations should also be considered in the context of our findings. Although weight and height data were prospectively collected, we relied on self-reported measures at ages 30 and 43. Even though a strong correlation has been found between self-reported and measured weight and height, self-report could be prone to underestimation, particularly in individuals with overweight or obesity [27]. This would have tended to bias our results towards the null. Whilst we used validated, self-report questionnaires to assess anxiety and depression, the GAD-7 and PHQ-9 are not diagnostic tools. Nevertheless, these instruments are widely used in epidemiological studies and scores greater than ten have previously demonstrated high sensitivity and specificity in detecting probable cases [11, 12]. Furthermore, it is important to note that the GAD-7 and PHQ-9 were only administered at one time point, so we did not have prior data in adolescents/young adulthood to determine the age of depression or anxiety onset, nor could we exclude the possibility of reverse causation.

Despite using a DAG to inform confounder adjustment, our simple causal diagram could not depict the complex interrelationships between a time-varying exposure and time-varying confounders. Additionally, several known risk factors identified by our DAG were not measured in the TAHS cohort and could not be adjusted for in our analyses. These included family history of depression and obesity, medication use, genetics, history of trauma or chronic stress and substance abuse. Consequently, our results might have some residual confounding. Attrition bias could be a possibility as men and those with obesity at age 43 were more likely to be lost to follow-up. However, no differences in the proportion of participants lost to follow up between BMI trajectories were seen. Moreover, the *persistently high* and *child average-increasing* trajectories had the smallest sample sizes and may have lacked precision, particularly in stratified analyses. Although the shape and size of our trajectories are comparable to prior research in the field [8], TAHS participants were born in 1961, more than a decade before the prevalence of childhood obesity began to notably increase in the community. Based on current trends, we would expect that present-day trajectories may differ in shape and size, with a greater proportion of participants belonging to the highest trajectories. Therefore, further research is necessary to quantify the effects of more recent trends in BMI trajectories from childhood. Lastly, multiple comparisons might have increased the risk of type 1 error, especially given that we considered *p*-values of the interaction terms to be significant at a level of 0.1. Therefore, the results of effect modification analyses should be interpreted with some caution.

## Conclusion

A persistently high BMI and a consistently increasing BMI over the life course was associated with an increased risk of depression in middle age, with members of the weight gain trajectory more likely to have increased severity of symptoms - although we can’t exclude the possible role of early-onset mental illness. Encouragingly, resolving early-life adiposity was associated with more favorable anxiety profiles and fewer depressive symptoms. These findings have important public health implications, as they highlight the need to target mental-health screening and treatment towards at-risk trajectory groups and emphasize the importance of early intervention to prevent and resolve excess weight. Further research is required to understand the causal, potentially bidirectional, or common mechanisms underlying these associations, and how these mechanisms could be addressed therapeutically. Furthermore, possible factors that might exacerbate or negate risks should be further explored, as these could be manipulated as part of clinical management.

## Supplementary information

Supplementary Material

## Data Availability

Individual participant data may be provided on request by anyone with a proposal. The proposal will be considered by the TAHS steering committee. Request can be directed to SCD (who is PI of TAHS and corresponding author of this paper). Data for all TAHS participants may be provided.
