# Supplementary material for: Life course BMI trajectories from childhood to mid-adulthood are differentially associated with anxiety and depression outcomes in middle age
Source: Int J Obes (Lond). 2023 May 9;47(8):661–8. doi: 10.1038/s41366-023-01312-6 (PMC10359183; doi:10.1038/s41366-023-01312-6)
Supplement: Supplementary file 1 — Supplementary Material [file 41366_2023_1312_MOESM1_ESM.docx]

**Life Course BMI Trajectories from Childhood to Mid-Adulthood Are Differentially Associated with Anxiety and Depression Outcomes in Middle Age**

Claire Gallagher^1^ MPH, Jane Pirkis^2^ PhD, Katrina Lambert^3^ PhD, Jennifer L. Perret^1^ PhD, Gulshan B. Ali^1^ MSc, Caroline J. Lodge^1^ PhD, Gayan Bowatte PhD^1,4^, Garun S. Hamilton^5,6^ PhD, Melanie C. Matheson PhD^1,7^, Dinh S. Bui^1^ PhD, Michael J. Abramson^8^ PhD, E. Haydn Walters MD DSc^1,9^ Shyamali C. Dharmage^1*^PhD, Bircan Erbas^3*^PhD.

* equal senior author

**Institutional Affiliation:**

^1^ Centre for Epidemiology and Biostatistics, Melbourne School of Population and Global Health, University of Melbourne, Melbourne Australia

^2^ Centre for Mental Health, Melbourne School of Population and Global Health, University of Melbourne, Melbourne Australia

^3^School of Psychology and Public Health, La Trobe University, Melbourne, Australia

^4^ Department of Basic Sciences, Faculty of Allied Health Sciences, University of Peradeniya, Peradeniya, Sri Lanka

^5^Department of Lung, Sleep, Allergy & Immunology, Monash Health, Melbourne, Australia

^6^School of Clinical Sciences, Monash University, Melbourne, Australia

^7^Population Health Solutions, Telstra Health, Melbourne, Victoria, Australia

^8^School of Public Health & Preventive Medicine, Monash University, Melbourne, Australia

^9^School of Medicine, University of Tasmania, Hobart, Australia

***E-methods*…………………………………………………………3**

***Supplementary Figure S1………………………………………..6***

***Supplementary Figure S2 ………………………………………..7***

***References*………………………………………………………….9**

***Table S1*……………………………………………………….......10**

***Table S2*……………………………………………………….......11**

***Table S3*……………………………………………………….......12**

***Table S4*……………………………………………………….......13**

***Table S5*……………………………………………………….......14**

***Table S6*……………………………………………………….......16**

***Table S7*………………………………………………….…….......18**

***Table S8*………………………………………………….…….......19**

***Table S9*……………………………………………………….........20**

***Table S10…*………………………………………………………...21**

**e-Methods:**

**Tasmanian Longitudinal Health Study**

This study analyzed data from the Tasmanian Longitudinal Health Study (TAHS), a population-based, prospective community birth cohort study of children born in 1961, attending school in Tasmania, Australia in 1968 when baseline data collection occurred. Details regarding TAHS sampling procedures and methods of data collection have previously been described.(1) In summary, n=8583 school children (age 7) underwent a clinical examination and questionnaire data were collected from their parents. Follow up surveys and/or examinations were conducted in 1974, 1979, 1991, 2002-2008 and 2012-2016 (Supplementary Figure S1). Demographic details, anthropometric measures and personal smoking data were collected at each time point. Questions assessing mental health and alcohol intake were included in the most recent survey. Data on this cohort has also been extracted from hospital birth and school medical records.

**Ethics Statement**

Baseline study (1968) and follow-ups in 1974 and 1979 were approved by the Tasmanian Minister of Health and the human ethics review committee at the University of Tasmania. The 2002, 2010, and 2012 follow-ups were approved by the human ethics review committees at the University of Melbourne (approval number 040375), Tasmania (040375.1) and New South Wales (08094), the Alfred Hospital (1118/04), and Royal Brisbane and Women's Hospital health service district (2006/037) and conducted following the amended Declaration of Helsinki. written informed consent was obtained from all participants.

**Outcomes**

***2.2.1 Depression***

Depression outcomes were measured via self-report at age 53 using the 9-item Patient Health Questionnaire (PHQ-9)(2). The PHQ-9 is a validated screening tool that was developed to briefly assess the diagnostic criteria for depressive disorders outlined in the fourth edition of the Diagnostic and Statistical Manual of Mental Disorders (DSM-IV), by asking participants how often in the previous two weeks, have they been bothered by the follow nine symptoms: (1) anhedonia; (2) depressed mood; (3) sleep disturbances; (4) fatigue; (5) appetite changes; (6) low self-esteem; (7) concentration problems; (8) psychomotor disturbances; and (9) passive thoughts of death or self-harm (2). For each symptom, participants rated the frequency of occurrence on a scale of 0 to 3 (0 = not at all, 1 = several days, 2 = more than half of the days and 3 = nearly every day) and each response was summed to generate a total PHQ-9 score ranging from 0 to 27. In this study, the recommended cutoff value of 10 was used to define participants as having prevalent depression, as previous research has shown that PHQ-9 scores ≥ 10 had a sensitivity and specificity of 88% for detecting major depression (2). In addition, we used cutoff points of 5, 10, 15 and 20 to define depression severity, with PHQ-9 scores ranging from 0-4, 5-9, 10-14, 15-19 and 20-27 representing “minimal”, “mild” “moderate” “moderately severe” and “severe” depression, respectively (2). Given the heterogenous nature of depression, we also assessed the 9 depressive symptoms as individual outcomes as different symptoms may have different risk factors (3).

***2.2.2 Anxiety***

Anxiety outcomes were measured via self-report at age 53 using the 7-item Generalized Anxiety Disorder scale (GAD-7), a validated tool designed to assess the key diagnostic criteria for generalized anxiety disorder outlined in the DSM-IV (4). Participants were asked how often in the previous two weeks, have they been bothered by symptoms of: (1) feeling nervous, anxious or on edge; (2) not being able to stop or control worrying; (3) worrying too much about different things; (4) having trouble relaxing; (5) being so restless that it is hard to sit still; (6) becoming easily annoyed or irritable; (7) Feeling afraid as if something awful might happen (4). For each symptom, participants rated the frequency of occurrence on a scale of 0 to 3 (0 = not at all, 1 = several days, 2 = more than half of the days and 3 = nearly every day) and each response was summed to generate a total GAD-7 score ranging from 0 to 21. A total GAD-7 score ≥ 10 has been shown to have good sensitivity (0.89) and specificity (0.82) in detecting those with anxiety and cut points of 5, 10 and 15 are considered to represent mild, moderate and severe levels of anxiety, respectively (4).

**Other variables**

***Smoking Status***

At age 53, smoking status was determined by asking participants “in your lifetime, have you smoked at least 100 cigarettes or equal amounts of cigars, pipes or any tobacco product?” and “do you currently smoke?”. Participants were categorized as “never smokers”, “former smokers” and “current smokers” accordingly.

***Occupational Class***

In accordance with the Australian Standard Classification of Occupations (ASCO) (5), participants were classified into the following five categories based on the skill level of their occupation: (1) managers, administrators and professionals; (2) associate professionals; (3) tradespersons and related workers, advanced clerical and service workers; (4) intermediate clerical, sales and service works, and intermediate production and transport workers; (5) elementary clerical, sales and service workers, laborers and related workers, including houseworkers.

***Educational Attainment***

Participants were categorized into the following three categories based on their level of educational attainment: (1) less than high school; (2) completion of high school, trade/apprenticeship, certificate, or diploma; (3) university or post graduate degree.

***Alcohol intake***

Beverage-specific alcohol intake was measured at age 53 using the Anti-Cancer Council Victoria Food Frequency Questionnaire (ACCV FFQ)(6). Participants were asked about their consumption frequency and quantity (in number of glasses) of full-strength beer, low-strength beer, red wine, white wine, fortified wine, and spirits in the previous 12 months. Using the NHMRC standard drinks guide (7), beverage-specific intake in glasses was converted to standard drinks based on the estimated ethanol content of each beverage (1.4 standard drinks (sd) per glass of full-strength beer; 0.8 sd per glass of low-strength beer; 1.6 sd per glad of red wine; 1.4 sd per glass of white wine; 0.8 sd per glass of fortified wine; and 1 sd per glass of spirits)l. Weekly alcohol intake (standard drinks per week) was then calculated by summing participant’s beverage-specific intakes and dividing the total by 52. Based on the NHMRC alcohol guidelines which recommend consuming fewer than 10 standard drinks per week to reduce the risk of alcohol related harm (7), participants were defined as “non-drinkers”, “low-risk drinkers” or “at risk drinkers” if they consumed <1 sd per week, ≥1 but <10 sd per week and ≥10 sd per week, respectively.

**Derivation of BMI trajectories using Group-Based Trajectory Modelling**

Group-based trajectory modelling (GBTM) was used to identify and define groups of participants following similar patterns of BMI across childhood and adulthood. Trajectories were modeled in STATA using the plug-in developed by Jones and Nagin(8). The sample for the analysis was restricted to those with BMI available for at least three-time points, of which two were from childhood and one from adulthood. BMI data were fitted to a censored normal model, with age in years as the measure of time. A stepwise approach was taken to determine the optimal number of trajectories and polynomial order. Initially, a one trajectory model with the highest order polynomial (quintic) was fitted to the dataset. If the polynomial was not significantly different from zero, the order of the polynomial was reduced until significance was reached (p<0.05). The number of trajectories was then increased by one and the steps were repeated until the highest order polynomial for each trajectory was significant. The optimal model was selected based on the Bayesian Information Criteria (BIC), group posterior probability and plausibility/clinical interpretation of trajectory shapes.

A five-trajectory model was chosen as it had the lowest BIC, an average posterior probability >0.81 and the shape of trajectories were comparable. The five trajectories were labelled according to their unique shapes: persistently average (50.3%; n=2109), persistently low (26.3%; n=1120), child high-decreasing (14.1%; n=592), child average-increasing (5.8%; n=245), and persistently high (3.1%; n=128).

**Supplementary Figure S1. Tasmanian Longitudinal Health Study follow up details**

**
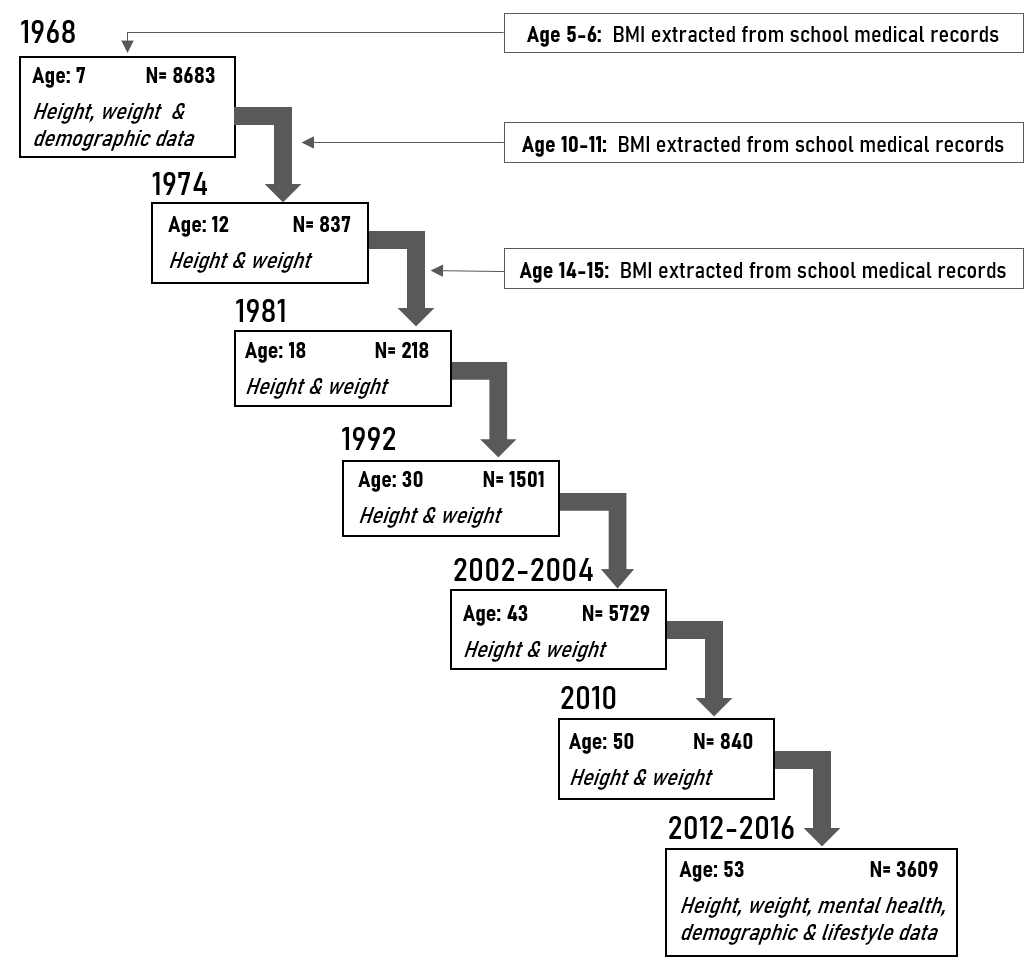
**

**Supplementary Figure S2. Directed Acyclic Graph of the Association Between BMI Trajectories and Mental Health at Age 53.**


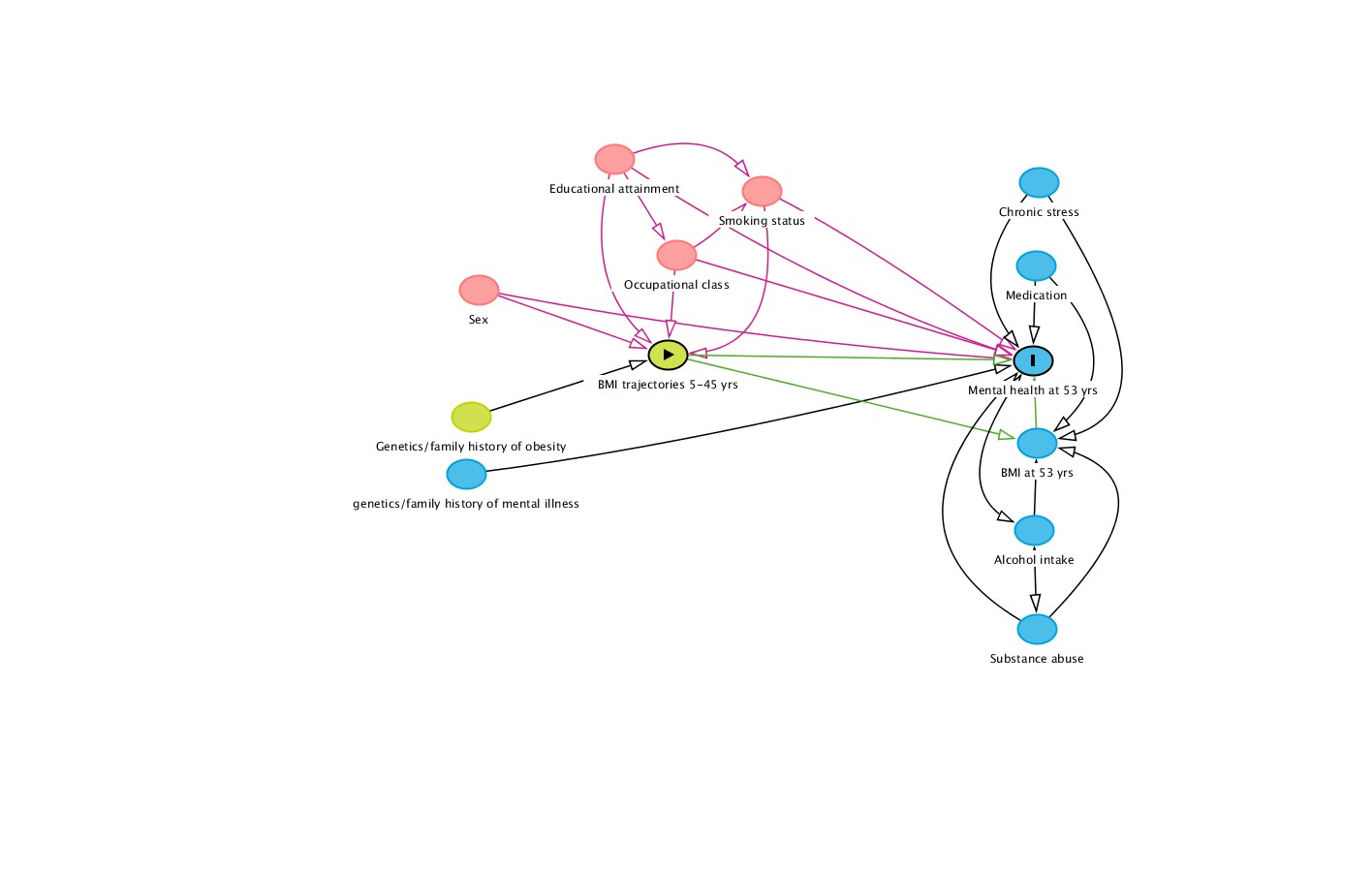


**References**

1. Matheson MC, Abramson MJ, Allen K, Benke G, Burgess JA, Dowty JG, et al. Cohort Profile: The Tasmanian Longitudinal Health STUDY (TAHS). Int J Epidemiol. 2017;46(2):407-8i.

2. Martin A, Rief W, Klaiberg A, Braehler E. Validity of the Brief Patient Health Questionnaire Mood Scale (PHQ-9) in the general population. Gen Hosp Psychiatry. 2006;28(1):71-7.

3. Fried EI, Nesse RM. Depression sum-scores don't add up: why analyzing specific depression symptoms is essential. BMC Med. 2015;13(1):72.

4. Spitzer RL, Kroenke K, Williams JB, Lowe B. A brief measure for assessing generalized anxiety disorder: the GAD-7. Arch Intern Med. 2006;166(10):1092-7.

5. Australian Bureau of Statistics. Australian Standard Classification of Occupations: Second Edition. Canberra: Australian Bureau of Statistics; 1997.

6. Hodge A, Patterson AJ, Brown WJ, Ireland P, Giles G. The Anti Cancer Council of Victoria FFQ: relative validity of nutrient intakes compared with weighed food records in young to middle-aged women in a study of iron supplementation. Aust N Z J Public Health. 2000;24(6):576-83.

7. National Health and Medical Research Council. Australian Guidelines to Reduce Health Risks from Drinking Alcohol. Australian Government; 2020.

8. Jones BL, Nagin DS. A Note on a Stata Plugin for Estimating Group-based Trajectory Models. Sociological Methods & Research. 2013;42(4):608-13.

| **Supplementary Table S1. Characteristics of participants loss to follow up** | | | |
| --- | --- | --- | --- |
| Characteristics | Retained from 43 to 53 years  (n = 2478) | Loss to follow up from 43 to 53 years  (n = 1716) | P Value |
| **BMI Trajectories -% (N)** |  |  | 0∙138 |
| Persistently Average | 59∙08% (1246) | 40∙92% (863) |  |
| Persistently Low | 60∙71% (680) | 39∙29% (440) |  |
| Child High-Decreasing | 59∙80 (354) | 40∙20% (238) |  |
| Child Average-Increasing | 52∙65% (129) | 47∙35% (116) |  |
| Persistently High | 53∙91% (69) | 46∙09% (59) |  |
| **Weight status at 43** |  |  | **<0∙001** |
| Normal/Underweight | 60∙72% (1096) | 39∙28% (709) |  |
| Overweight | 60.46% (948) | 39∙54% (620) |  |
| Obese | 52∙86% (434) | 47∙14% (387) |  |
| **Sex** |  |  | **<0∙001** |
| Male | 56∙29% (1213) | 43∙71% (942) |  |
| Female | 62∙04% (1265) | 37∙96% (774) |  |
| BMI, body mass index. P value derived from chi-square test | | | |

| **Supplementary Table S2. Distribution of depression and anxiety outcomes across BMI trajectory groups stratified by sex** | | | | | | | |
| --- | --- | --- | --- | --- | --- | --- | --- |
| **Females** | **Total**  **(n = 1240)** | **Persistently Average**  **(n = 599)** | **Persistently Low**  **(n = 338)** | **Child High-Decreasing**  **(n = 185)** | **Child Average-Increasing**  **(n = 77)** | **Persistently High**  **(n = 41)** | **P-Value** |
| **Current Depression– n (%)** | 165  (9∙2%) | 42  (7∙1%) | 29  (8∙8%) | 14  (7∙4%) | 13  (17∙1%) | 6  (14∙3%) | **0∙02** |
| **Severity of Depressive Symptoms – n (%)** |  |  |  |  |  |  | **<0∙01** |
| Minimal | 1320 (73∙8%) | 455 (76∙5%) | 249 (75∙9%) | 147  (77∙8%) | 43  (56∙6%) | 30  (71∙4%) |  |
| Mild | 303 (17∙0%) | 98  (16∙5%) | 50 (15∙2%) | 28  (14∙8%) | 20  (26∙3%) | 6  (14∙3%) |  |
| Moderate | 99  (5∙5%) | 31  (5∙2%) | 16  (4∙9%) | 8  (4∙2%) | 4  (5∙3%) | 5  (11∙9%) |  |
| Moderately severe | 40  (2∙2%) | 7  (1∙2%) | 10  (3∙1%) | 5  (2∙7%) | 4  (5∙3%) | 1  (2∙4%) |  |
| Severe | 26  (1∙5%) | 4  (0∙6%) | 3  (0∙9%) | 1  (0∙5%) | 5  (6∙6%) | 0  (0∙0%) |  |
| **Current Anxiety– n(%)** |  |  |  |  |  |  | 0∙57 |
| Yes | 210  (11∙6%) | 61  (10∙2∙%) | 34  (10∙1%) | 16  (8∙6%) | 12  (15∙6%) | 4  (9∙8%) |  |
| **Severity of Anxiety Symptoms** |  |  |  |  |  |  | 0∙74 |
| Minimal | 1230  (68∙1%) | 419  (70∙0%) | 230 (68∙1%) | 133  (71∙9%) | 47  (61∙0%) | 29  (70∙7%) |  |
| Mild | 365  (20∙2%) | 465  (77∙6%) | 257 (76∙0%) | 142  (76∙8%) | 52  (67∙53%) | 34  (82∙9%) |  |
| Moderate | 133  (7∙4%) | 82  (13∙7%) | 51 (15∙01%) | 27  (14∙6%) | 14  (18∙2%) | 4  (9∙8%) |  |
| Severe | 77  (4∙3%) | 33  (5∙5%) | 17  (5∙0%) | 8  (4∙3%) | 7  (9∙1%) | 3  (7∙3%) |  |
| **Males** | **Total**  **(n=1188)** | **Persistently Average**  **(n = 618)** | **Persistently Low**  **(n = 332)** | **Child High-Decreasing**  **(n = 160)** | **Child Average-Increasing**  **(n = 51)** | **Persistently**  **High**  **(n = 27)** | **P-Value** |
| **Current Depression– n (%)** | 90  (5∙2%) | 30  (4∙9%) | 17  (5∙1%) | 6  (3∙7%) | 4  (8∙2%) | 5  (19∙2%) | **0∙02** |
| **Severity of Depressive Symptoms – n (%)** |  |  |  |  |  |  | **0∙02** |
| Minimal | 1373  (79∙9%) | 499  (80∙9%) | 271  (81∙4%) | 128  (79∙5%) | 32  (65∙3%) | 18  (69∙2%) |  |
| Mild | 256% (14∙9%) | 88  (14∙3%) | 45  (13∙5%) | 27  (16∙8%) | 13  (26∙5%) | 3  (11∙5%) |  |
| Moderate | 54  (3∙1%) | 20  (3∙2%) | 10  (3∙0%) | 5  (3∙1%) | 3  (6∙1%) | 2  (7∙7%) |  |
| Moderately severe | 18  (1∙01%) | 4  (0∙6%) | 4  (1∙2%) | 0  (0∙0%) | 0  (0∙0%) | 2  (7∙7%) |  |
| Severe | 18  (1∙01%) | 6  (1∙0%) | 3  (0∙9%) | 1  (0∙6%) | 1  (2∙0%) | 1  (3∙9%) |  |
| **Current Anxiety– n(%)** | 116  (6∙7%) | 39  (6∙3%) | 18  (5∙4%) | 8  (5∙00%) | 4  (7∙8%) | 3  (11∙1%) | 0∙71 |
| **Severity of Anxiety Symptoms** |  |  |  |  |  |  | 0∙18 |
| Minimal | 1331  (76∙8%) | 497  (80∙4%) | 273  (82∙2%) | 139  (86∙9%) | 38  (74∙5%) | 18  (66∙7) |  |
| Mild | 287  (16∙6%) | 88  (14∙2%) | 43  (13∙0%) | 14  (8∙8%) | 9  (17∙7%) | 7  (25∙9%) |  |
| Moderate | 70  (4∙0%) | 15  (2∙4%) | 10  (3∙0%) | 5  (3∙1%) | 1  (2∙0%) | 0  (0∙0%) |  |
| Severe | 46  (2∙7%) | 18  (2∙9%) | 6  (1∙8%) | 2  (1∙3%) | 3  (5∙9%) | 2  (7∙1%) |  |
| BMI, body mass index. P value derived from chi-square test | | | | | | | |

| **Supplementary Table S3. Distribution of the frequency of depressive symptoms for the total sample and by sex** | | | | |
| --- | --- | --- | --- | --- |
| **Symptoms of Depression** | **Total** | **Females** | **Males** | **p-value of sex differences** |
| **Anhedonia - %(n)** | n=3594 | n=1834 | n=1760 | 0∙03 |
| Not at all | 75∙79% (2724) | 77∙15% (1415) | 74∙38% (1309) |  |
| Several days | 18∙17% (653) | 16∙52% (303) | 19∙89% (350) |  |
| More than half the days | 3∙90% (140) | 3∙82% (70) | 3∙98% (70) |  |
| Nearly every day | 2∙14% (77) | 2∙51% (46) | 1∙76% (31) |  |
| **Depressed mood - %(n)** | n=3586 | N=1830 | N=1756 | 0∙11 |
| Not at all | 76∙66% (2749) | 75∙30 (1378) | 78∙08% (1371) |  |
| Several days | 17∙90% (642) | 18∙47% (338) | 17∙31% (304) |  |
| More than half the days | 2∙93% (105) | 3∙28% (60) | 2∙56% (45) |  |
| Nearly every day | 2∙51% (90) | 2∙95% (54) | 2∙05% (36) |  |
| **Sleep disturbances - %(n)** | n=3588 | N=1833 | N=1755 | <0∙001 |
| Not at all | 52∙54% (1885) | 46∙64% (855) | 58∙69% (1030) |  |
| Several days | 30∙71% (1102) | 33∙22% (609) | 28∙09% (493) |  |
| More than half the days | 8∙70% (312) | 10∙04% (184) | 7∙29% (128) |  |
| Nearly every day | 8∙05% (289) | 10∙09% (185) | 5∙93% (104) |  |
| **Fatigue - %(n)** | n=3568 | N=1820 | N=1748 | <0∙001 |
| Not at all | 44∙37% (1583) | 39∙62% (721) | 49∙31% (862) |  |
| Several days | 40∙55% (1447) | 43∙08% (784) | 37∙93% (663) |  |
| More than half the days | 8∙38% (299) | 9∙07% (165) | 7∙67% (134) |  |
| Nearly every day | 6∙70% (239) | 8∙24% (150) | 5∙09% (89) |  |
| **Appetite changes - %(n)** | n=3573 | N=1822 | N=1751 | <0∙001 |
| Not at all | 72∙18% (2579) | 67∙40% (1228) | 77∙16% (1351) |  |
| Several days | 19∙26% (687) | 21∙84% (398) | 16∙50% (289) |  |
| More than half the days | 5∙37% (192) | 6∙64% (121) | 4∙05% (71) |  |
| Nearly every day | 3∙22% (115) | 4∙12% (75) | 2∙28% (40) |  |
| **Low self-esteem - %(n)** | n=3585 | N=1832 | N=1753 |  |
| Not at all | 82∙65% (2963) | 79∙37% (1454) | 86∙08% (1509) | <0∙001 |
| Several days | 12∙50% (448) | 14∙52% (266) | 10∙38% (182) |  |
| More than half the days | 2∙48% (89) | 2∙89% (53) | 2∙05% (36) |  |
| Nearly every day | 2∙37% (85) | 3∙22% (59) | 1∙48% (26) |  |
| **Concentration problems - %(n)** | n=3595 | N=1835 | N=1760 | 0∙17 |
| Not at all | 81∙39% (2926) | 80∙11% (1470) | 82∙73% (82∙73) |  |
| Several days | 13∙35% (480) | 14∙06% (258) | 12∙61% (222) |  |
| More than half the days | 2∙78% (100) | 2∙94% (54) | 2∙61% (46) |  |
| Nearly every day | 2∙48% (89) | 2∙89% (53) | 2∙05% (36) |  |
| **Psychomotor disturbances - %(n)** | n=3598 | N=1836 | N=1762 | 0∙15 |
| Not at all | 92∙69% (3335) | 91∙88% (1687) | 93∙53% (1648) |  |
| Several days | 4∙84% (174) | 5∙50% (101) | 4∙14% (73) |  |
| More than half the days | 1∙25% (45) | 1∙47% (27) | 1∙02% (18) |  |
| Nearly every day | 1∙22% (44) | 1∙14% (21) | 1∙31% (23) |  |
| **Suicidal ideation - %(n)** | n=3598 | N=1836 | N=1762 |  |
| Not at all | 96∙19% (3461) | 96∙08% (1764) | 96∙31% (1697) | 0∙18 |
| Several days | 2∙81% (101) | 2∙67% (49) | 2∙95% (52) |  |
| More than half the days | 0∙56% (20) | 0∙82% (15) | 0∙28% (5) |  |
| Nearly every day | 0∙44% (16) | 0∙44% (8) | 0∙45% (8) |  |
| BMI, body mass index. P value derived from chi-square test | | | | |

| **Supplementary Table S4. Distribution of the frequency of anxiety symptoms for the total sample and by sex** | | | | |
| --- | --- | --- | --- | --- |
| **Anxiety Symptoms** | **Total** | **Females** | **Males** | **p-value of sex differences** |
| **Feeling nervous/anxious - %(n)** | N=3591 | N=1832 | N=1759 | <0∙001 |
| Not at all | 59∙31% (2130) | 53∙17% (974) | 65∙72% (1156) |  |
| Several days | 29∙02% (1402) | 32∙70% (599) | 25∙18% (443) |  |
| More than half the days | 6∙07% (218) | 7∙10% (130) | 5∙00% (88) |  |
| Nearly every day | 5∙60% (201) | 7∙04% (129) | 4∙09% (72) |  |
| **Uncontrollable worrying - %(n)** | N=3589 | N=1834 | N=1755 | <0∙001 |
| Not at all | 69∙52% (2495) | 64∙29% (1179) | 74∙99% (1316) |  |
| Several days | 20∙73% (744) | 23∙66% (434) | 17∙66% (310) |  |
| More than half the days | 5∙27% (189) | 6∙16% (113) | 4∙33% (76) |  |
| Nearly every day | 4∙49% (161) | 5∙89% (108) | 3∙02% (53) |  |
| **Excessive worrying - %(n)** | N=3588 | N=1833 | N=1755 | <0∙001 |
| Not at all | 54∙93% (1971) | 50∙68% (959) | 59∙37% (1042) |  |
| Several days | 33∙11% (1188 | 35∙08% (643) | 31∙05% (545) |  |
| More than half the days | 6∙05% (217) | 6∙71% (123) | 5∙36% (94) |  |
| Nearly every day | 5∙91% (212) | 7∙53% (138) | 4∙22% (74) |  |
| **Trouble relaxing - %(n)** | N=3578 | N=1827 | N=1751 | <0∙001 |
| Not at all | 61∙96% (2217) | 57∙42% (1049) | 66∙70% (1168) |  |
| Several days | 25∙94% (928) | 28∙08% (513) | 23∙70% (415) |  |
| More than half the days | 6∙82% (244) | 7∙72% (141) | 5∙88% (103) |  |
| Nearly every day | 5∙28% (189) | 6∙79% (124) | 3∙71% (65) |  |
| **Restlessness - %(n)** | N=3582 | N=1830 | N=1752 | 0∙25 |
| Not at all | 76∙86% (2753) | 75∙63% (1384) | 78∙14% (1369) |  |
| Several days | 15∙05% (539) | 15∙68% (287) | 14∙38% (252) |  |
| More than half the days | 4∙55% (163) | 5∙08% (93) | 4∙00% (70) |  |
| Nearly every day | 3∙55% (127) | 3∙61% (66) | 3∙48% (61) |  |
| **Irritability - %(n)** | N=3588 | N=1832 | N=1756 | 0∙06 |
| Not at all | 57∙25% (2054) | 55∙29% (1013) | 59∙28% (1041) |  |
| Several days | 33∙82% (1194) | 34∙99% (641) | 31∙49% (553) |  |
| More than half the days | 5∙94% (213) | 5∙79% (106) | 6∙09% (107) |  |
| Nearly every day | 3∙54% (127) | 3∙93% (72) | 3∙13% (55) |  |
| **Feelings of apprehension - %(n)** | N=3589 | N=1831 | N=1758 | <0∙001 |
| Not at all | 83∙87% (3010) | 80∙94% (1482) | 86∙92% (1528) |  |
| Several days | 11∙17% (401) | 12∙89% (236) | 9∙39% (165) |  |
| More than half the days | 2∙73% (98) | 3∙17% (58) | 2∙28% (40) |  |
| Nearly every day | 2∙23% (80) | 3∙00% (55) | 1∙42% (25) |  |
| BMI, body mass index. P value derived from chi-square test | | | | |

| **Supplementary Table S5. Distribution of potential effect modifiers across mental health outcomes** | | | | | | |
| --- | --- | --- | --- | --- | --- | --- |
|  | **Depression**  **(PHQ9 ≥10)** | | **p-value** | **Anxiety**  **(GAD7 ≥10)** | | **p-value** |
|  | **Yes**  **(n = 255)** | **No**  **(n = 3252)** |  | **Yes**  **(n = 326)** | **No**  **(n = 3213)** |  |
| **Sex- % (n)** |  |  | <0∙001 |  |  | <0∙001 |
| Male | 94∙76% (1629) | 5∙24% (90) |  | 6∙69% (116) | 93∙31% (1618) |  |
| Female | 90∙77% (1623) | 9∙23% (165) |  | 11∙63% (210) | 88∙37% (1595) |  |
| **Alcohol Intake -**% (n) |  |  | <0∙001 |  |  | <0∙01 |
| None | 11∙36% (102) | 88∙64% (796) |  | 11∙98% (109) | 88∙02% (801) |  |
| <10 standard drinks/week | 5∙91% (75) | 94∙09% (1194) |  | 8∙38% (107) | 91∙62% (1170) |  |
| ≥10 standard drinks/week | 5∙76% (77) | 94∙24% (1260) |  | 8∙15% (110) | 91∙85% (1240) |  |
| **Smoking Status -**% (n) |  |  | <0∙001 |  |  | <0∙001 |
| Never | 5∙50% (84) | 94∙50% (1442) |  | 7∙13% (109) | 92∙87% (1419) |  |
| Former | 6∙68% (90) | 93∙32 (1258) |  | 8∙92% (122) | 91∙08% (1245) |  |
| Current | 12∙77% (76) | 87∙23% (519) |  | 14∙85% (90) | 85∙15% (516) |  |
| BMI, body mass index. P value derived from chi-square test | | | | | | |

| **Supplementary Table S6. Univariable binary and ordinal logistic regression exploring the association between life course BMI trajectory groups and mental health outcomes at age 53** | | | | | | | | |
| --- | --- | --- | --- | --- | --- | --- | --- | --- |
|  | **Persistently Low**‡  **(n= 615)** | | **Child High-Decreasing**‡  **(n= 330)** | | **Child Average-increasing**‡  **(n= 125)** | | **Persistently High**‡  **(n= 68)** | |
| **Symptoms of Depression  ^b^** | OR  (95% CI) | P | OR  (95% CI) | P | OR  (95% CI) | P | OR  (95% CI) | P |
| Anhedonia | 0∙88  (0∙70,1∙10) | 0∙25 | 0∙93  (0∙70,1∙23) | 0∙61 | 1∙72  (1∙17,2∙52) | **<0∙01** | 1∙30  (0∙76,2∙21) | 0∙33 |
| Depressed mood | 0∙94  (0∙75,1∙17) | 0∙58 | 0∙73  (0∙54,0∙99) | **0∙04** | 1∙70  (1∙16, 2∙50) | **<0∙01** | 0∙94  (0∙52, 1∙69) | 0∙85 |
| Sleep disturbances | 1∙08  (0∙90,1∙29) | 0∙40 | 0∙80  (0∙64,1∙01) | **0∙06** | 1∙88  (1∙35,2∙62) | **<0∙01** | 1∙22  (0∙77,1∙93) | 0∙40 |
| Fatigue | 1∙03  (0∙87,1∙24) | 0∙71 | 0∙90  (0∙72,1∙12) | 0∙34 | 1∙84  (1∙31,2∙60) | **<0∙01** | 1∙56  (0∙99,2∙47) | 0∙06 |
| Appetite changes | 0∙70  (0∙56,0∙87) | **<0∙01** | 1∙04  (0∙80,1∙34) | 0∙78 | 2∙42  (1∙70,3∙46) | **<0∙01** | 2∙20  (1∙37,3∙52) | **<0∙01** |
| Low self-esteem | 0∙96  (0∙75,1∙24) | 0∙77 | 1∙14  (0∙84,1∙55) | 0∙41 | 2∙14  (1∙42,3∙21) | **<0∙01** | 1∙75  (1∙00,3∙09) | **0∙05** |
| Concentration problems | 0∙93  (0∙73,1∙19) | 0∙57 | 1∙03  (0∙76,1∙40) | 0∙83 | 2∙03  (1∙36,3∙03) | **<0∙01** | 1∙51  (0∙86,2∙65) | 0∙15 |
| Psychomotor disturbances | 1∙03  (0∙70,1∙51) | 0∙87 | 1∙27  (0∙81,2∙00) | 0∙29 | 1∙55  (0∙82,2∙94) | 0∙18 | 1∙19  (0∙47,3∙05) | 0∙71 |
| Suicidal ideation | 1∙38  (0∙85,2∙25) | 0∙20 | 1∙37  (0∙75,2∙51) | 0∙31 | 2∙89  (1∙44,5∙80) | **<0∙01** | 2∙92  (1∙19,7∙14) | **0∙02** |
|  |  |  |  |  |  |  |  |  |
| **Current Depression ^a^** | 1∙18  (0∙81,1∙74) | 0∙39 | 0∙96  (0∙58,1∙60) | 0∙87 | 2∙49  (1∙42, 4∙38) | **<0∙01** | 3∙06  (1∙54, 6∙08) | **<0∙01** |
| **Severity of Depressive Symptoms  ^b^** |  |  |  |  |  |  |  |  |
| Minimal  Mild  Moderate  Moderately Severe  Severe | 1∙02  (0∙81,1∙28) | 0∙87 | 1∙01  (0∙75,1∙34) | 0∙97 | 2∙49  (1∙71,3∙62) | **<0∙01** | 1∙71  (1∙00,2∙92) | **0∙05** |
|  | **Persistently Low**‡  **(n=670)** | | **Child High-Decreasing**‡  **(n=345)** | | **Child Average-increasing**‡  **(n=128)** | | **Persistently High**‡  **(n=68)** | |
| **Symptoms of Anxiety ^b^** | OR  (95% CI) | P | OR  (95% CI) | P | OR  (95% CI) | P | OR  (95% CI) | P |
| Feeling nervous/anxious | 1∙02  (0∙85,1∙23) | 0∙85 | 0∙86  (0∙67,1∙09) | 0∙21 | 1∙49  (1∙05,2∙10) | **0∙03** | 1∙03  (0∙63,1∙66) | 0∙92 |
| Uncontrollable worrying | 1∙05  (0∙86,1∙28) | 0∙64 | 0∙94  (0∙73,1∙22) | 0∙65 | 1∙60  (1∙11,2∙32) | **0∙01** | 1∙25  (0∙76,2∙06) | 0∙38 |
| Excessive worrying | 1∙05  (0∙87,1∙26) | 0∙62 | 0∙98  (0∙7,1∙23) | 0∙84 | 1∙40  (0∙99,1∙98) | **0∙05** | 1∙08  (0∙68,1∙72) | 0∙76 |
| Trouble relaxing | 1∙05  (0∙87,1∙27) | 0∙61 | 1∙07  (0∙87,1∙27) | 0∙61 | 1∙37  (0∙97,1∙97) | 0∙08 | 1∙03  (0∙63,1∙67) | 0∙92 |
| Restlessness | 0∙91  (0∙73,1∙14) | 0∙42 | 0∙82  (0∙62,1∙10) | 0∙18 | 1∙05  (0∙69,1∙60) | 0∙82 | 1∙10  (0∙64,1∙90) | 0∙71 |
| Irritability | 0∙93  (0∙77,1∙12) | 0∙44 | 0∙96  (0∙76,1∙21) | 0∙71 | 1∙74  (1∙24,2∙45) | **<0∙01** | 1∙61  (1∙02,2∙54) | **0∙04** |
| Feelings of apprehension | 0∙85  (0∙65,1∙12) | 0∙23 | 0∙84  (0∙59,1∙18) | 0∙30 | 1∙84  (1∙21,2∙82) | **0∙01** | 1∙26  (0∙68,2∙33) | 0∙47 |
|  |  |  |  |  |  |  |  |  |
| **Current Anxiety ^a^** | 0∙94  (0∙66,1∙33) | 0∙73 | 0∙84  (0∙53,1∙33) | 0∙45 | 1∙60  (0∙91,2∙80) | 0∙10 | 1∙28  (0∙57,2∙88) | 0∙55 |
| **Severity of Anxiety Symptoms ^b^** |  |  |  |  |  |  |  |  |
| Minimal  Mild  Moderate  Severe | 0∙99  (0∙79,1∙25) | 0∙96 | 0∙87  (0∙64,1∙17) | 0∙35 | 1∙62  (1∙09,2∙41) | **0∙02** | 1∙15  (0∙65,2∙94) | 0∙63 |
|  | **Persistently Low**‡  **(n= 654)** | | **Child High-Decreasing**‡  **(n342)** | | **Child Average-increasing**‡  **(n=124)** | | **Persistently High**‡  **(n=67)** | |
| **Severity of Depression/Anxiety Symptoms ^b^** |  |  |  |  |  |  |  |  |
| Minimal  Mild  Moderate  Severe | 0∙98  (0∙78,1∙23) | 0∙86 | 091  (0∙68,1∙22) | 0∙53 | 1∙78  (1∙20,2∙64) | **<0∙01** | 1∙37  (0∙79,2∙38) | 0∙26 |
| BMI, body mass index.  ‡ *persistently average* trajectory is the reference group  ^a^ Binary logistic regression  ^b^ Ordinal logistic regression | | | | | | | | |

| **Supplementary Table S7. Univariable binary and ordinal logistic regression exploring the association between BMI trajectory groups and depression and anxiety outcomes at age 53, stratified by sex** | | | | | | | | | |
| --- | --- | --- | --- | --- | --- | --- | --- | --- | --- |
|  | **Persistently Low** ‡  **(n= 654)** | | **Child High-Decreasing** ‡  **(n= 342)** | | **Child Average-increasing** ‡  **(n= 124)** | | **Persistently High** ‡  **(n= 67)** | |  |
|  | Female  (n=326) | Male  (n=328) | Female  (n=185) | Male  (n=157) | Female  (n=75) | Male  (n=49) | Female  (n=41) | Male  (n=26) | **LTR**  **p-value** |
| **Symptoms of Depression ^b^** |  |  |  |  |  |  |  |  |  |
| Anhedonia | 0∙91  (0∙65,1∙26) | 0∙86  (0∙63,1∙16) | 1∙06  (0∙71,1∙57) | 0∙83  (0∙56,1∙23) | 1∙66  (0∙98,2∙79) | 1∙93*  (1∙09,3∙14) | 0∙98  (0∙46,2∙09) | 2∙01  (0∙92,4∙38) | 0∙59 |
| Depressed mood | 1∙05  (0∙77,1∙43) | 0∙84  (0∙61,1∙15) | 0∙81  (0∙54,1∙21) | 0∙66  (0∙42,1∙02) | 2∙15**  (1∙33,3∙50) | 1∙14  (0∙59,2∙19) | 0∙79  (0∙361∙74) | 1∙22  (0∙51,2∙96) | 0∙42 |
| Sleep disturbances | 1∙21  (0∙95,1∙56) | 0∙95  (0∙73,1∙23) | 0∙81  (0∙60,1∙11) | 0∙75  (0∙53,1∙06) | 1∙87**  (1∙22,2∙86) | 1∙70*  (1∙00,2∙91) | 0∙98  (0∙55,1∙76) | 1∙53  (0∙72,3∙27) | 0∙57 |
| Fatigue | 1∙26  (0∙98,1∙62) | 0∙85  (0∙66,1∙09) | 1∙01  (0∙75,3∙31) | 0∙76  (0∙55,1∙06) | 2∙13**  (1∙36,3∙31) | 1∙41  (0∙82,2∙45) | 1∙60  (0∙89,2∙86) | 1∙43  (0∙67,3∙02) | 0∙22 |
| Appetite changes | 0∙70*  (0∙52,0∙94) | 0∙69*  (0∙50,0∙96) | 1∙08  (0∙77,1∙52) | 0∙95  (0∙64,1∙41) | 2∙38**  (1∙50,3∙77) | 2∙32**  (1∙32,4∙07) | 2∙01*  (1∙11,3∙65) | 2∙26*  (1∙04,4∙90) | 0∙99 |
| Low self-esteem | 0∙81  (0∙58,1∙15) | 1∙18  (0∙81,1∙73) | 1∙11  (0∙75,1∙65) | 1∙13  (0∙70,1∙85) | 2∙14**  (1∙29,3∙55) | 1∙87  (0∙93,3∙79) | 1∙43  (0∙70,2∙90) | 2∙19  (0∙85,5∙61) | 0∙59 |
| Concentration problems | 1∙02  (0∙72,1∙43) | 0∙84  (0∙59,1∙20) | 1∙01  (0∙67,1∙54) | 1∙05  (0∙68,1∙64) | 2∙15**  (1∙27,3∙61) | 1∙86*  (0∙99,3∙47) | 1∙76  (0∙88,3∙52) | 1∙11  (0∙41,2∙98) | 0∙87 |
| Psychomotor disturbances | 0∙99  (0∙58,1∙67) | 1∙08  (0∙62,1∙89) | 1∙33  (0∙74,2∙39) | 1∙17  (0∙58,2∙36) | 1∙39  (0∙60,3∙21) | 1∙77  (0∙66,4∙71) | 1∙05  (0∙31,3∙55) | 1∙39  (0∙32, 6∙10) | 0∙98 |
| Suicidal ideation | 1∙43  (0∙72,2∙85) | 1∙33  (0∙66,2∙67) | 1∙40  (0∙60,3∙25) | 1∙34  (0∙56,3∙23) | 3∙14*  (1∙28,7∙73) | 2∙56  (0∙84,7∙79) | 1∙58  (0∙35,7∙01) | 5∙15*  (1∙63,16∙21) | 0∙75 |
|  |  |  |  |  |  |  |  |  |  |
| **Current Depression ^a^** | 1∙28  (0∙78,2∙09) | 1∙05  (0∙57,1∙94) | 1∙05  (0∙56,1∙97) | 0∙76  (0∙31,1∙85) | 2∙72**  (1∙38,5∙33) | 1∙74  (0∙59,5∙15) | 2∙19  (0∙88,5∙50) | 4∙66**  (1∙64,13∙21) | 0∙66 |
|  |  |  |  |  |  |  |  |  |  |
| **Severity of Depressive Symptoms ^b^** |  |  |  |  |  |  |  |  |  |
| Minimal  Mild  Moderate  Moderately Severe  Severe | 1∙06  (0∙77,1∙45) | 0∙97  (0∙69,1∙37) | 0∙94  (0∙64,1∙39) | 1∙07  (0∙70,1∙64) | 2∙16**  (1∙62,4∙22) | 2∙17*  (1∙183∙99) | 1∙38  (0∙69,2∙74) | 2∙24  (0∙94,5∙32) | 0∙83 |
|  |  |  |  |  |  |  |  |  |  |
| **Symptoms of Anxiety ^b^** |  |  |  |  |  |  |  |  |  |
| Feeling nervous/anxious | 1∙16  (0∙90,1∙50) | 0∙87  (0∙66,1∙14) | 0∙96  (0∙70,1∙32) | 0∙70  (0∙48,1∙02) | 1∙37  (0∙87,2∙15) | 1∙54  (0∙89,2∙67) | 0∙82  (0∙44,1∙52) | 1∙32  (0∙61,2∙85) | 0∙28 |
| Uncontrollable worrying | 1∙15  (0∙88,1∙50) | 0∙92  (0∙68, 1∙25) | 0∙89  (0∙63,1∙26) | 0∙98  (0∙66,1∙45) | 1∙41  (0∙88,2∙26) | 1∙75  (0∙96,3∙19) | 0∙85  (0∙44,1∙66) | 2∙00  (0∙93,4∙29) | 0∙28 |
| Excessive worrying | 1∙15  (0∙89,1∙48) | 0∙94  (0∙72, 1∙22) | 1∙07  (0∙78,1∙47) | 0∙85  (0∙60,1∙20) | 1∙43  (0∙92,2∙24) | 1∙27  (0∙74,2∙20) | 0∙85  (0∙46,1∙56) | 1∙46  (0∙70, 3∙04) | 0∙48 |
| Trouble relaxing | 1∙25  (0∙97,1∙63) | 0∙85  (0∙65, 1∙13) | 1∙31  (0∙95,1∙80) | 0∙81  (0∙57,1∙17) | 1∙48  (0∙94,2∙32) | 1∙17  (0∙66,2∙06) | 0∙74  (0∙38,1∙43) | 1∙57  (0∙76, 3∙26) | **0∙05** |
| Restlessness | 0∙99  (0∙73,1∙34) | 0∙83  (0∙60,1∙15) | 0∙99  (0∙68,1∙44) | 0∙63*  (0∙40, 1∙00) | 1∙06  (0∙61,1∙83) | 1∙02  (0∙52,2∙00) | 0∙73  (0∙33,1∙59) | 1∙81  (0∙83,3∙95) | 0∙21 |
| Irritability | 1∙00  (0∙77,1∙30) | 0∙86  (0∙66, 1∙13) | 0∙98  (0∙71,1∙36) | 0∙93  (0∙66,1∙30) | 1∙60*  (1∙02,2∙51) | 1∙98*  (1∙16,3∙37) | 1∙29  (0∙70,2∙36) | 2∙19*  (1∙08,4∙43) | 0∙58 |
| Feelings of apprehension | 0∙98  (0∙69,1∙39) | 0∙68  (0∙45, 1∙04) | 1∙01  (0∙66,1∙55) | 0∙58  (0∙32, 1∙06) | 1∙86*  (1∙09,3∙20) | 1∙69  (0∙84,3∙41) | 0∙64  (0∙24,1∙65) | 2∙66*  (1∙14,6∙20) | **0∙05** |
|  |  |  |  |  |  |  |  |  |  |
| **Current Anxiety ^a^** | 0∙99  (0∙63,1∙54) | 0∙94  (0∙66,1∙33) | 0∙83  (0∙47,1∙49) | 0∙83  (0∙53,1∙33) | 1∙63  (0∙83,3∙18) | 1∙60  (0∙91,2∙80) | 0∙95  (0∙33,2∙77) | 1∙28  (0∙89,4∙14) | 0∙91 |
|  |  |  |  |  |  |  |  |  |  |
| **Severity of Anxiety Symptoms ^b^** |  |  |  |  |  |  |  |  |  |
| Minimal  Mild  Moderate  Severe | 1∙08  (0∙82,1∙44) | 0∙79  (0∙58,1∙08) | 0∙91  (0∙63,1∙30) | 0∙55*  (0∙35,0∙87) | 1∙52  (0∙94,2∙45) | 1∙39  (0∙76,2∙53) | 0∙95  (0∙48,1∙88) | 1∙92  (0∙89,4∙14) | 0∙15 |
|  |  |  |  |  |  |  |  |  |  |
| **Severity of Depression/Anxiety Symptoms ^b^** |  |  |  |  |  |  |  |  |  |
| Minimal  Mild  Moderate  Severe | 1∙02  (0∙75,1∙29) | 0∙93  (0∙65,1∙31) | 0∙98  (0∙67,1∙44) | 0∙76  (0∙47,1∙23) | 1∙62  (0∙98,2∙70) | 1∙86  (0∙98,3∙54) | 0∙93  (0∙45,1∙95) | 2∙21  (0∙97,5∙14) | 0∙35 |
| ‡ reference category is the *persistently average* trajectory group.  The P value is derived from likelihood ratio tests.  ^a^ binary logistic regression  ^b^ ordered logistic regression  *<0.05; **<0.01; *** <0∙001 | | | | | | | | | |

| **Supplementary Table S8. Multivariable ordinal logistic regression models exploring the association between BMI trajectory groups and depression and anxiety symptoms at age 53** | | | | | | | | |
| --- | --- | --- | --- | --- | --- | --- | --- | --- |
|  | **Persistently Low**‡  **(n= 615)** | | **Child High-Decreasing**‡  **(n= 330)** | | **Child Average-increasing**‡  **(n= 125)** | | **Persistently High**‡  **(n= 68)** | |
|  | OR  (95% CI) | P | OR  (95% CI) | P | OR  (95% CI) | P | OR  (95% CI) | P |
| **Depression Symptoms** |  |  |  |  |  |  |  |  |
| Anhedonia | 0∙89  (0∙70,1∙12) | 0∙30 | 0∙90  (0∙67,1∙20) | 0∙46 | 1∙83  (1∙24,2∙72) | **<0∙01** | 1∙30  (0∙75,2∙25) | 0∙35 |
| Depressed mood | 0∙97  (0∙77, 1∙22) | 0∙76 | 0∙67  (0∙49, 0∙92) | **0∙01** | 1∙62  (1∙09,2∙41) | **0∙02** | 0∙84  (0∙46,1∙56) | 0∙59 |
| Sleep disturbances | 1∙09  (0∙90,1∙31) | 0∙37 | 0∙74  (0∙59, 0∙94) | **0∙01** | 1∙71  (1∙22, 2∙42) | **<0∙01** | 1∙14  (0∙72, 1∙83) | 0∙57 |
| Fatigue | 1∙04  (0∙87, 1∙25) | 0∙65 | 0∙85  (0∙68,1∙07) | 0∙17 | 1∙75  (1∙23, 2∙49) | **<0∙01** | 1∙50  (0∙95, 2∙39) | 0∙09 |
| Appetite changes | 0∙71  (0∙56, 0∙89) | **<0∙01** | 0∙95  (0∙72, 1∙24) | 0∙69 | 2∙26  (1∙56, 3∙27) | **<0∙01** | 2∙14  (1∙32, 3∙45) | **<0∙01** |
| Low self-esteem | 1∙02  (0∙79, 1∙33) | 0∙86 | 1∙07  (0∙78, 1∙47) | 0∙68 | 2∙03  (1∙33, 3∙09) | **<0∙01** | 1∙62  (0∙90, 2∙91) | 0∙11 |
| Concentration problems | 0∙98  (0∙76, 1∙26) | 0∙86 | 0∙95  (0∙69, 1∙30) | 0∙73 | 1∙93  (1∙27, 2∙91) | **<0∙01** | 1∙62  (0∙91, 2∙87) | 0∙10 |
| Psychomotor disturbances | 1∙08  (0∙73, 1∙60) | 0∙70 | 1∙11  (0∙69, 1∙80) | 0∙66 | 1∙41  (0∙72, 2∙76) | 0∙32 | 1∙20  (0∙46, 3∙13) | 0∙70 |
| Suicidal ideation | 1∙41  (0∙85, 2∙34) | 0∙19 | 1∙22  (0∙64, 2∙33) | 0∙55 | 3∙15  (1∙55, 6∙42) | **<0∙01** | 2∙47  (0∙92, 6∙61) | 0∙07 |
| **Anxiety Symptoms** |  |  |  |  |  |  |  |  |
| Feeling nervous/anxious | 1∙03  (0∙85, 1∙25) | 0∙76 | 0∙81  (0∙30, 1∙03) | 0∙09 | 1∙38  (0∙96, 1∙97) | 0∙08 | 0∙89  (0∙54, 1∙46) | 0∙64 |
| Uncontrollable worrying | 1∙07  (0∙87, 1∙31) | 0∙54 | 0∙88  (0∙67, 1∙15) | 0∙36 | 1∙44  (0∙98, 2∙11) | 0∙06 | 1∙04  (0∙62, 1∙75) | 0∙89 |
| Excessive worrying | 1∙04  (0∙86, 1∙25) | 0∙69 | 0∙93  (0∙73, 1∙18) | 0∙54 | 1∙29  (0∙90, 1∙84) | 0∙16 | 0∙96  (0∙59, 1∙56) | 0∙88 |
| Trouble relaxing | 1∙06  (0∙87, 1∙28) | 0∙58 | 0∙99  (0∙78, 1∙27) | 0∙95 | 1∙28  (0∙89, 1∙84) | 0∙19 | 0∙91  (0∙55, 1∙50) | 0∙70 |
| Restlessness | 0∙91  (0∙73, 1∙15) | 0∙44 | 0∙76  (0∙56, 1∙02) | 0∙07 | 1∙02  (0∙66, 1∙57) | 0∙93 | 1∙12  (0∙64, 1∙95) | 0∙70 |
| Irritability | 0∙95  (0∙78, 1∙15) | 0∙60 | 0∙91  (0∙71, 1∙15) | 0∙42 | 1∙86  (1∙31, 2∙64) | **<0∙01** | 1∙52  (0∙95, 2∙44) | 0∙08 |
| Feelings of apprehension | 0∙84  (0∙63, 1∙10) | 0∙20 | 0∙76  (0∙53, 1∙09) | 0∙13 | 1∙67  (1∙07, 2∙59) | **0∙02** | 1∙08  (0∙56, 2∙06) | 0∙82 |
| **Severity of Depression/Anxiety Symptoms** |  |  |  |  |  |  |  | 0∙38 |
| Minimal  Mild  Moderate  Severe | 1∙01  (0∙80, 1∙28) | 0∙92 | 0∙83  (0∙61, 1∙13) | 0∙23 | 1∙67  (1∙10, 2∙52) | **0∙02** | 1∙29  (0∙73, 2∙27) |  |
| BMI, body mass index.  ‡ reference category is the *persistently average* trajectory group.  Adjusted for sex, educational attainment, occupational status, smoking status | | | | | | | | |

| **Supplementary Table S9. Multivariable ordinal logistic regression models exploring the association between BMI trajectory groups and depression and anxiety symptoms at age 53, stratified by sex** | | | | | | | | | |
| --- | --- | --- | --- | --- | --- | --- | --- | --- | --- |
|  | **Persistently Low**‡  **(n= 615)** | | **Child High-Decreasing**‡  **(n= 330)** | | **Child Average-increasing**‡  **(n= 125)** | | **Persistently High**‡  **(n= 68)** | |  |
|  | **Female**  **(n=328)** | **Male**  **(n=333)** | **Female**  **(n=189)** | **Male**  **(n=161)** | **Female**  **(n=76)** | **Male**  **(n=49)** | **Female**  **(n=42)** | **Male**  **(n=26)** |  |
|  | **OR**  **(95%CI)** | **OR**  **(95%CI)** | **OR**  **(95%CI)** | **OR**  **(95%CI)** | **OR**  **(95%CI)** | **OR**  **(95%CI)** | **OR**  **(95%CI)** | **OR**  **(95%CI)** | **LTR**  **p-value** |
| **Depression Symptoms** |  |  |  |  |  |  |  |  |  |
| Anhedonia | 0∙96  (0∙68,1∙35) | 0∙81  (0∙59,1∙11) | 0∙99  (0∙66,1∙50) | 0∙78  (0∙52,1∙18) | 1∙68  (0∙98,2∙86) | **2∙11***  **(1∙17,3∙81)** | 0∙92  (0∙42,2∙00) | 2∙04  (0∙90,4∙62) | 0∙58 |
| Depressed mood | 1∙08  (0∙78,1∙48) | 0∙86  (0∙61,1∙20) | 0∙73  (0∙48,1∙11) | **0∙61***  **(0∙38,0∙99)** | **1∙94****  **(1∙18,3∙20)** | 1∙21  (0∙61,2∙39) | 0∙73  (0∙33,1∙64) | 1∙07  (0∙41,2∙76) | 0∙66 |
| Sleep disturbances | 1∙26  (0∙98,1∙62) | 0∙93  (0∙71,1∙22) | **0∙73***  **(0∙53,1∙00)** | 0∙73  (0∙51,1∙05) | **1∙82****  **(1∙18,2∙81)** | 1∙60  (0∙91,2∙81) | 1∙03  (0∙57,1∙86) | 1∙36  (0∙63,2∙95) | 0∙56 |
| Fatigue | ***1∙29****  ***(1∙00,1∙67)*** | 0∙83  (0∙64,1∙08) | 0∙97  (0∙71,1∙32) | 0∙72  (0∙51,1∙02) | **2∙04****  **(1∙30,3∙20)** | 1∙42  (0∙80,2∙50) | 1∙72  (0∙95,3∙09) | 1∙25  (0∙59,2∙69) | 0∙19 |
| Appetite changes | **0∙71***  **(0∙52,0∙97)** | 0∙70*  (0∙49,0∙99) | 1∙03  (0∙73,1∙46) | 0∙84  (0∙55,1∙29) | **2∙33*****  **(1∙46,3∙73)** | **2∙25****  **(1∙23,4∙12)** | **2∙14****  **(1∙17,3∙91)** | 2∙05  (0∙92,4∙58) | 0∙97 |
| Low self-esteem | 0∙89  (0∙62,1∙26) | 1∙24  (0∙84,1∙83) | 1∙07  (0∙71,1∙60) | 1∙06  (0∙63,1∙79) | **2∙02****  **(1∙20,3∙40)** | 2∙03  (0∙96,4∙26) | 1∙48  (0∙72,3∙04) | 1∙91  (0∙69,5∙29) | 0∙76 |
| Concentration problems | 1∙04  (0∙74,1∙48) | 0∙90  (0∙62,1∙31) | 0∙94  (0∙61,1∙45) | 0∙93  (0∙58,1∙51) | **1∙93***  **(1∙13,3∙31)** | **1∙97***  **(1∙02,3∙78)** | 1∙82  (0∙90,3∙68) | 1∙28  (0∙46,3∙51) | 0∙95 |
| Psychomotor disturbances | 1∙05  (0∙61,1∙80) | 1∙10  (0∙62,1∙97) | 1∙28  (0∙70,2∙37) | 0∙92  (0∙41,2∙05) | 1∙30  (0∙55,3∙05) | 1∙63  (0∙54,4∙89) | 1∙13  (0∙33,3∙86) | 1∙39  (0∙30,6∙39) | 0∙94 |
| Suicidal ideation | 1∙66  (0∙82,3∙38) | 1∙23  (0∙59,2∙58) | 1∙21  (0∙49,2∙98) | 1∙22  (0∙48,3∙13) | **3∙46****  **(1∙36,8∙82)** | 2∙81  (0∙91,8∙75) | 1∙70  (0∙37,7∙81) | **3∙92***  **(1∙05,14∙9)** | 0∙88 |
| **Anxiety Symptoms** |  |  |  |  |  |  |  |  |  |
| Feeling nervous/anxious | 1∙19  (0∙92,1∙54) | 0∙86  (0∙65,1∙14) | 0∙93  (0∙67,1∙28) | 0∙66*  (0∙45,0∙97) | 1∙27  (0∙80, 2∙02) | 1∙57  (0∙89,2∙79) | 0∙76  (0∙40,1∙42) | 1∙21  (0∙54,2∙70) | 0∙25 |
| Uncontrollable worrying | 1∙20  (0∙91,1∙58) | 0∙91  (0∙66,1∙25) | 0∙82  (0∙58,1∙18) | 0∙95  (0∙63,1∙43) | 1∙33  (0∙82,2∙15) | 1∙70*  (0∙91,3∙20) | 0∙75  (0∙37,1∙50) | 1∙69  (0∙76,3∙74) | 0∙27 |
| Excessive worrying | 1∙16  (0∙90,1∙51) | 0∙90  (0∙69,1∙19) | 1∙01  (0∙73, 1∙40) | 0∙81  (0∙57,1∙16) | 1∙32  (0∙84, 2∙08) | 1∙25  (0∙71,2∙20) | 0∙79  (0∙42, 1∙49) | 1∙31  (0∙61,2∙79) | 0∙50 |
| Trouble relaxing | ***1∙29***  ***(0∙99,1∙69)*** | 0∙82*  (0∙62,1∙09) | ***1∙18***  ***(0∙85,1∙63)*** | 0∙77  (0∙53,1∙11) | 1∙41  (0∙89,2∙24) | 1∙16  (0∙64,2∙11) | 0∙65  (0∙32,1∙29) | 1∙45  (0∙68,3∙11) | **0∙05** |
| Restlessness | 0∙99  (0∙72,1∙36) | 0∙84  (0∙60, 1∙17) | 0∙88  (0∙60,1∙31) | 0∙59*  (0∙36,0∙96) | 1∙01  (0∙58,1∙77) | 1∙05  (0∙52,2∙11) | 0∙74  (0∙33,1∙65) | 1∙89  (0∙85,4∙19) | 0∙26 |
| Irritability | 1∙05  (0∙80,1∙37) | 0∙86  (0∙65, 1∙13) | 0∙91  (0∙65,1∙26) | 0∙91  (0∙64, 1∙29) | 1∙59*  (1∙00,2∙52) | 2∙38**  (1∙37,4∙14) | 1∙23  (0∙66,2∙29) | 2∙02  (0∙98,4∙16) | 0∙40 |
| Feelings of apprehension | 1∙00  (0∙70,1∙43) | 0∙63*  (0∙40,0∙97) | 0∙94  (0∙60,1∙46) | 0∙51*  (0∙27,0∙95) | 1∙71  (0∙99,2∙98) | 1∙68  (0∙80,3∙51) | ***0∙61***  ***(0∙23,1∙62)*** | 2∙15  (0∙87,5∙35) | 0∙07 |
| **Severity of Depression/Anxiety Symptoms** |  |  |  |  |  |  |  |  | 0∙54 |
| Minimal  Mild  Moderate  Severe | 1∙08  (0∙79,1∙48) | 0∙93  (0∙65,1∙33) | 0∙90  (0∙61,1∙33) | 0∙69  (0∙42,1∙15) | 1∙61  (0∙96,2∙71) | 1∙85  (0∙94,3∙65) | 0∙92  (0∙44,1∙96) | 2∙08  (0∙86,5∙02) |  |
| The reference category is the *persistently average* trajectory group.  *<0.05; **<0.01; *** <0∙001  *Numbers in italics indicate p-value of the interaction <0∙01*  LTR P value is derived from likelihood ratio tests.  Adjusted for sex, educational attainment, occupational status, smoking status | | | | | | | | | |

| **Supplementary Table S10. Multivariable binary logistic regression investigating the modifying effect of sociodemographic and lifestyle covariates at age 53 on the relationship between BMI trajectories and current depression and current anxiety** | | | | | |
| --- | --- | --- | --- | --- | --- |
| **Effect Modifier Variables** | **Odds Ratio (95% CI) for Current Depression** | | | | |
|  | **Persistently Low**‡  **(n= 661)** | **Child High-Decreasing**‡  **(n= 350)** | **Child Average-increasing**‡  **(n= 125)** | **Persistently High**‡  **(n= 68)** | **LRT p-value** |
| **Occupational class** |  |  |  |  | 0∙09 |
| Skill Level 1 to 3 | 0∙98 (0∙59, 1∙63) | 0∙61 (0∙29, 1∙27) | 1∙50 (0∙61, 3∙71) | 0∙51 (0∙07, 3∙85) |  |
| Skill Level 4 and 5 | 1∙63 (0∙86, 3∙09) | 1∙23 (0∙53, 2∙87) | 3∙76 ** (1∙65, 8∙60) | ***5∙56*** (2∙27, 13∙63)*** |  |
|  | **Odds Ratio (95% CI) for Current Anxiety** | | | | |
| **Alcohol Intake** |  |  |  |  | *‡* |
| None | 0∙45 (0∙20, 1∙01) | 0∙93 (0∙42, 2∙09) | 1∙48 (0∙66, 3∙30) | 1∙80 (0∙62, 5∙18) |  |
| <10 standard drinks/week | ***1∙23 (0∙67, 2∙25)*** | 0∙67 (0∙27, 1∙67) | 1∙70 (0∙60, 4∙81) | 0∙95 (0∙20, 4∙39) |  |
| ≥10 standard drinks/week | ***1∙26 (0∙72, 2∙20)*** | 0∙76 (0∙32, 1∙77) | 0∙59 (0∙08, 4∙63) | (empty) |  |
| **Occupational class** |  |  |  |  | 0∙08 |
| Skill Level 1 to 3 | 1∙23 (0∙80, 1∙91) | 0∙64 (0∙33, 1∙25) | 1∙16 (0∙48, 2∙83) | 0∙43 (0∙06, 3∙26) |  |
| Skill Level 4 and 5 | ***0∙64 (0∙34, 1∙21)*** | 1∙01 (0∙49, 2∙09) | 2∙06 (0∙95, 4∙47) | 1∙84 (0∙70, 4∙79) |  |
| The reference category is the *persistently average* trajectory group.  *<0.05; **<0.01; *** <0∙001  *Numbers in italics indicate p-value of the interaction <0∙01*  LTR P value is derived from likelihood ratio tests.  ‡ likelihood ratio test unable compute due to empty cells or omitted data  Adjusted for sex, educational attainment, occupational class (alcohol model only), smoking status | | | | | |
